# Epigenetic and transcriptional dysregulation of *VWA2* associated with a *MYC*-driven oncogenic program in colorectal cancer

**DOI:** 10.1038/s41598-018-29378-7

**Published:** 2018-07-23

**Authors:** Beatriz González, Ferran Fece de la Cruz, Johanna Kristina Samuelsson, Andreu Alibés, Sergio Alonso

**Affiliations:** 1 Program of Predictive and Personalized Medicine of Cancer, Germans Trias i Pujol Research Institute, (IGTP-PMPPC), Campus Can Ruti, 08916 Badalona, Barcelona, Spain; 2 Active Motif, Inc, Carlsbad, CA 92008 USA; 3 Present Address: Massachusetts General Hospital Cancer Center and Department of Medicine, Harvard Medical School, Boston, MA USA

## Abstract

*VWA2* encodes AMACO, a secreted protein up-regulated in most colorectal carcinomas (CRC), constituting a promising biomarker. The mechanism responsible for its aberrant up-regulation has not been previously described. In this work, we analyzed *VWA2* DNA methylation in over 400 primary CRCs. No epigenetic alterations were found in its promoter-associated CpG island. However, the region located downstream of the transcriptional start site was hypomethylated in most CRCs. ChIP-Seq revealed increased levels of the active mark H3K4me3 and reduction of the repressive mark H3K27me3. In contrast, several CRC cell lines exhibited hypermethylation of *VWA2*. 5-AZA-2-deoxycitidine treatment led to transcriptional activation of *VWA2*, supporting a functional link between DNA methylation and transcription. *VWA2* expression in primary CRCs correlated with that of Myc and Myc-target genes. Transcriptional up-regulation of *VWA2* is extremely frequent (78%) and strong (average fold change >15) in CRC, but not in other types of cancer. *VWA2* undergoes hypomethylation in the majority of CRCs. This alteration could partly underlie the previously reported over-expression of AMACO. Co-expression profiling suggests that *VWA2* might be a constituent of a larger oncogenic transcriptional program regulated by c-Myc. Up-regulation of *VWA2* is virtually exclusive of CRC, reinforcing its potential as a specific biomarker.

## Introduction

The human gene *VWA2*, located in chromosome 10q25.3, encodes the von Willebrand factor A domain-containing protein 2 (Uniprot Q5GFL6, also known as AMACO)^1,2^. The primary sequence of AMACO manifests its extracellular localization: a signal peptide at the N-terminus is followed by a VWA domain, a cysteine-rich domain, an epidermal growth factor (EGF)-like domain with fully elongated O-glucosylation and O-fucosylation glycan chains, two more VWA domains, and another EGF-like domain (Fig. S1)^2,3^.

AMACO is expressed in a variety of cell types, including chondrocytes, keratinocytes and lung and uterine epithelial cells^2^. Its physiological functions, however, have just begun to be elucidated. Based on its cellular localization and domain structure, it has been postulated that AMACO participates in cell adhesion, migration, homing and signaling following ligand activation through its VWA domains^2^. AMACO also contains a RGD motif putatively involved in cell adhesion signaling through interaction with integrins^4,5^.

In a zebrafish study, AMACO was found to be a component of the Fraser complex, involved in the interaction between epithelial cells and the basal membranes. Mutations in *FRAS1*, *FREM2* and *GRIP1* genes, other known members of the extracellular Fraser complex, result on the autosomal recessive disorder known as Fraser Syndrome^6^, albeit no human pathological mutations in *VWA2* have been described to date. Double immunoelectron microscopy labeling on P0 mouse skin showed that Fras1 and AMACO co-localized in distinct regions, named anchoring plaques, in the dermis close to the basal membrane. Based on these observations, it has been suggested that the functions of AMACO in tumors somehow mimic its role during development, inducing matrix remodeling and signaling that might promote metastases^7^.

*VWA2* has a very low mutation rate in cancer, and no particular mutation hot-spot has been identified in its sequence according to COSMIC database (http://cancer.sanger.ac.uk/cosmic/gene/analysis?ln=VWA2)^8^. In colorectal cancer (CRC), a fusion mRNA involving the gene *TCF7L2* and exons 1 to 4 of *VWA2* has been identified. The reading frame of the parental genes is maintained in the fusion transcript keeping the potential to generate a new fusion protein of undetermined functionality^9^.

In 2005 AMACO was found to be aberrantly up-regulated in about 80% of human colorectal cancers (CRC), and in the majority of the colonic adenomas. Consequently, this protein was named colorectal cancer secreted protein 2 (CCSP-2)^10^. Since AMACO is an extracellular-matrix protein that can be secreted into the blood stream, detection of AMACO in plasma was proposed as a candidate serological biomarker of colon neoplasia, and specific immunodetection methods have been developed based on AMACO overexpression^11^.

Despite the putative interest of AMACO as a CRC biomarker, very little is known about the mechanism leading to its deregulation in colon cancer cells. In 2006 we reported the comprehensive analysis of DNA methylation alterations in a series of 149 gastrointestinal tumors using methylation sensitive amplified fragment length polymorphism (MS-AFLP), a fingerprinting technique to identify somatic changes in methylation at randomly selected *Not*I sites (5′GCGGCCGC3′) throughout the genome^12–14^. Using that technique, we identified a *Not* site undergoing somatic hypomethylation in around 15% of the colorectal cancers (CRC) and around 8% of the gastric cancers (GC). In this work, we further explored that initial observation, we mapped the frequently altered *Not*I site within the intronic region 635 bp downstream of the canonical transcriptional start site (TSS) of *VWA2* gene, and characterized in much higher detail the incidence, extension, and functional consequences of somatic DNA hypomethylation of *VWA2* in both primary CRC samples and cell lines. We also performed a comparative analysis of *VWA2* overexpression in 36 different types of cancer, exploring the data from the The Cancer Genome Atlas (TCGA).

## Results

### The first intron of *VWA2* is hypomethylated in primary colorectal cancers

**Figure 1:**
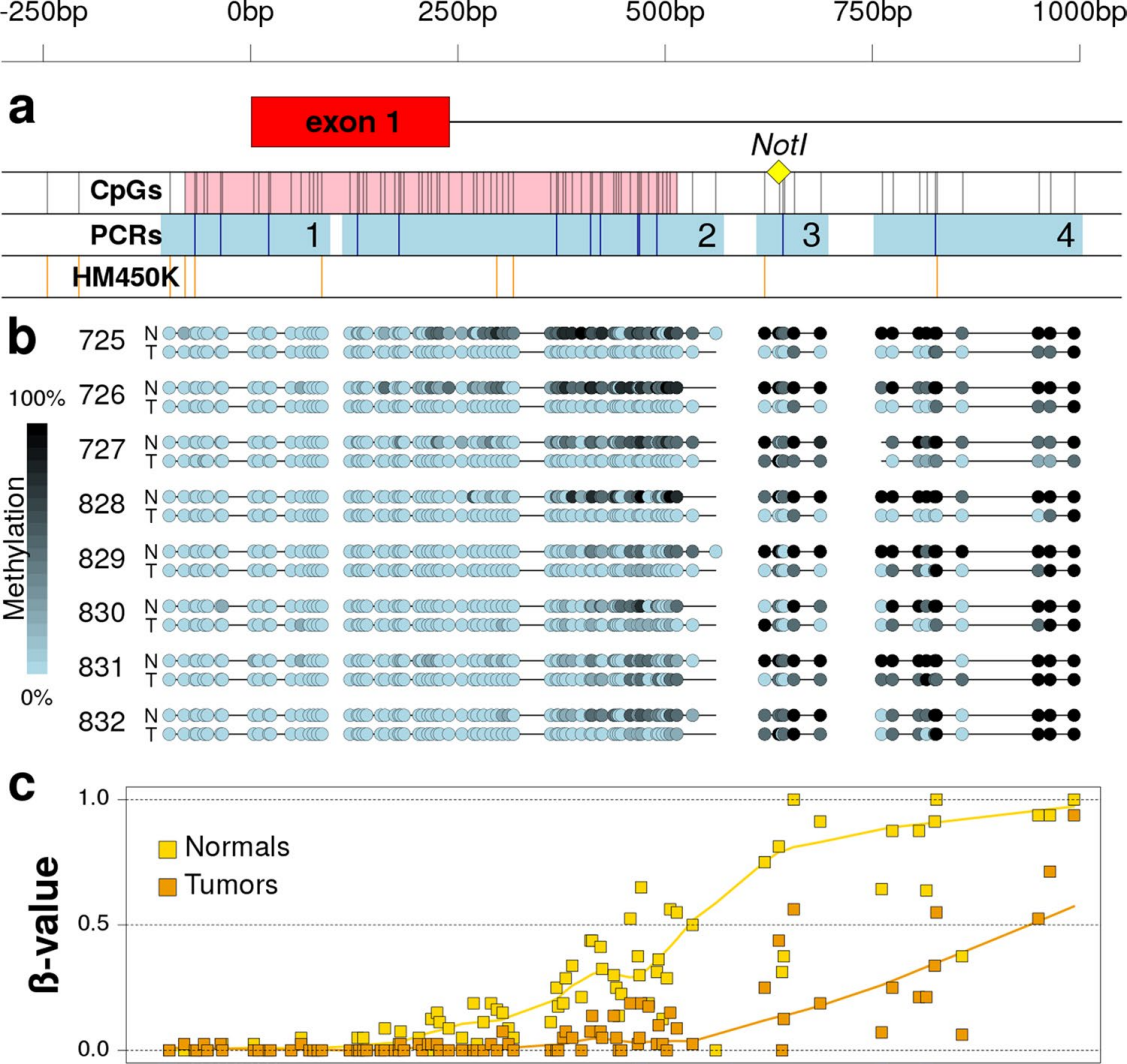
Methylation of the 5′-associated region of *VWA2* in normal and tumor tissues of CRC patients. The scale on top is valid for the three panels and refers to the distance (in bp) to the *VWA2* transcriptional start site. Panel a: Scheme of the DNA region containing the first exon of *VWA2* (exon 1, in red). The yellow diamond indicates the location of the *Not*I site originally found hypomethylated by the MS-AFLP analyses. The first track (CpGs) shows the CpG sites (black vertical bars) and the promoter-associated CpG island (in pink). The second track (PCRs) shows the four products analyzed by bisulfite-based PCR (light blue squares) and the *Bst*UI sites studied by COBRA (dark blue vertical bars). The third track (HM450K) indicates the position of the 10 probes of the Illumina HM450K methylation arrays within this region. Panel b: methylation status in normal (N) and tumor (T) tissues from CRC patients 725, 726, 727, 828, 829, 830, 831 and 832. Methylation was estimated by direct Sanger’s sequencing of the four PCR products shown in panel a. Panel c: average methylation level of normal (yellow) and tumor (orange) tissues from the eight patients shown in panel b, at every CpG site within the studied region. The solid lines represent the locally weighted moving average.

In a previous study, we employed MS-AFLP to profile somatic methylation alterations in 64 colorectal cancers and 85 gastric cancers, revealing the existence of a hypomethylated genomic *locus* corresponding to an electrophoretic band named C-46^13^. That band was excised from a MS-AFLP gel, re-amplified by PCR using the NotI and Mse + C primers (Supplementary Table ST1), and sequenced. The resultant sequence mapped at a *Not*I site located 637 bp downstream of the transcriptional start site (TSS) of the gene *VWA2*, outside the CpG island (CGI) that completely covers its first exon (Fig. 1a).

We extended the number of CRC cases analyzed by MS-AFLP to a total of 81. The *Not*I site was considered to be somatically hypomethylated in 14 cases (17%, scoring methodology detailed in methods). No significant association was found between somatic hypomethylation of this *Not*I site and patient gender, race, tumor location, MSI phenotype or the presence of mutations in *TP53*, *KRAS* or *BRAF*. We found, however, a positive association with patient age (*P* = 0.007, Student’s t-test, Table 1). This association retained statistical significance in multifactorial logistic regression analysis including age, gender, race, tumor location, MSI status and mutations in *TP53*, *KRAS* and *BRAF* as explanatory factors (OR = 1.12 per year, CI = 1.04–1.23, *P* = 0.005, Supplementary Table ST2).

**Table 1:** Clinico-pathological characteristics of 81 CRC cases classified according to *VWA2* methylation analyzed by MS-AFLP.

		No Somatic Hypomethylation	Somatic Hypomethylation	*P*-value
Total		67 (83%)	14 (17%)	
Gender	Female	30 (79%)	8 (21%)	0.56
	Male	37 (86%)	6 (14%)	
Race	Caucasian	40 (83%)	8 (17%)	0.76
	African Amer.	21 (81%)	5 (19%)	
Age		63.6 ± 10.7	74.3 ± 12.1	**0.007**
Tumor	Proximal	38 (84%)	7 (16%)	0.57
Location	Distal	27 (79%)	7 (21%)	
MSI status	MSS	58 (83%)	12 (17%)	1.0
	MSI	8 (80%)	2 (2%)	
*TP53*	Wild Type	31 (84%)	6 (16%)	1.0
	Mutant	29 (85%)	5 (15%)	
*KRAS*	Wild Type	40 (87%)	6 (13%)	0.52
	Mutant	22 (81%)	5 (19%)	
*BRAF*	Wild Type	50 (85%)	9 (15%)	0.61
	Mutant	6 (75%)	2 (25%)	

In parentheses, the percentage of cases without or with somatic hypomethylation within each studied group (in rows). *P*-values were calculated by Fisher’s exact test except for patient age, where Student’s t-test was applied. In bold, *P*-values < 0.01.

We analyzed the CpG methylation levels around the TSS of *VWA2* by bisulfite direct-PCR sequencing in a subset of colorectal cancer samples. The analyzed sequence comprised its first exon, the associated CpG island and the north and south CpG island shores (Fig. 1b). Since this region was too long for a single bisulfite-PCR reaction (1.1 Kb, encompassing 89 CpG sites), the analyses were performed in 4 separate PCRs (regions 1 to 4, Fig. 1a). No substantial somatic differences in methylation were found in the first exon or the area close to the TSS, that were essentially unmethylated in both normal and tumor tissues. Downstream of the first exon, however, the difference in methylation between normal and tumor samples gradually increased along the south shore of the CGI, were the MS-AFLP-detected *Not*I site was located (Fig. 1b,c). *Bst*UI-based combined bisulfite and restriction analyses (COBRA)^15^ of these four regions was employed to further validate these observations in the samples previously analyzed by MS-AFLP. No clear changes in the COBRA electrophoretic profiles between normal and tumor tissues were found in PCR regions 1 and 2. COBRA detected somatic changes in methylation in region 3, although in some samples the results were difficult to interpret due to the intermediate levels of methylation in both normal and tumor tissues (not shown). The PCR to analyze the region 4 was 315 bp containing a single *Bst*UI site (Fig. 1a), and clearly confirmed the hypomethylation of this region in tumors (Supplementary Fig. S2).

**Figure 2:**
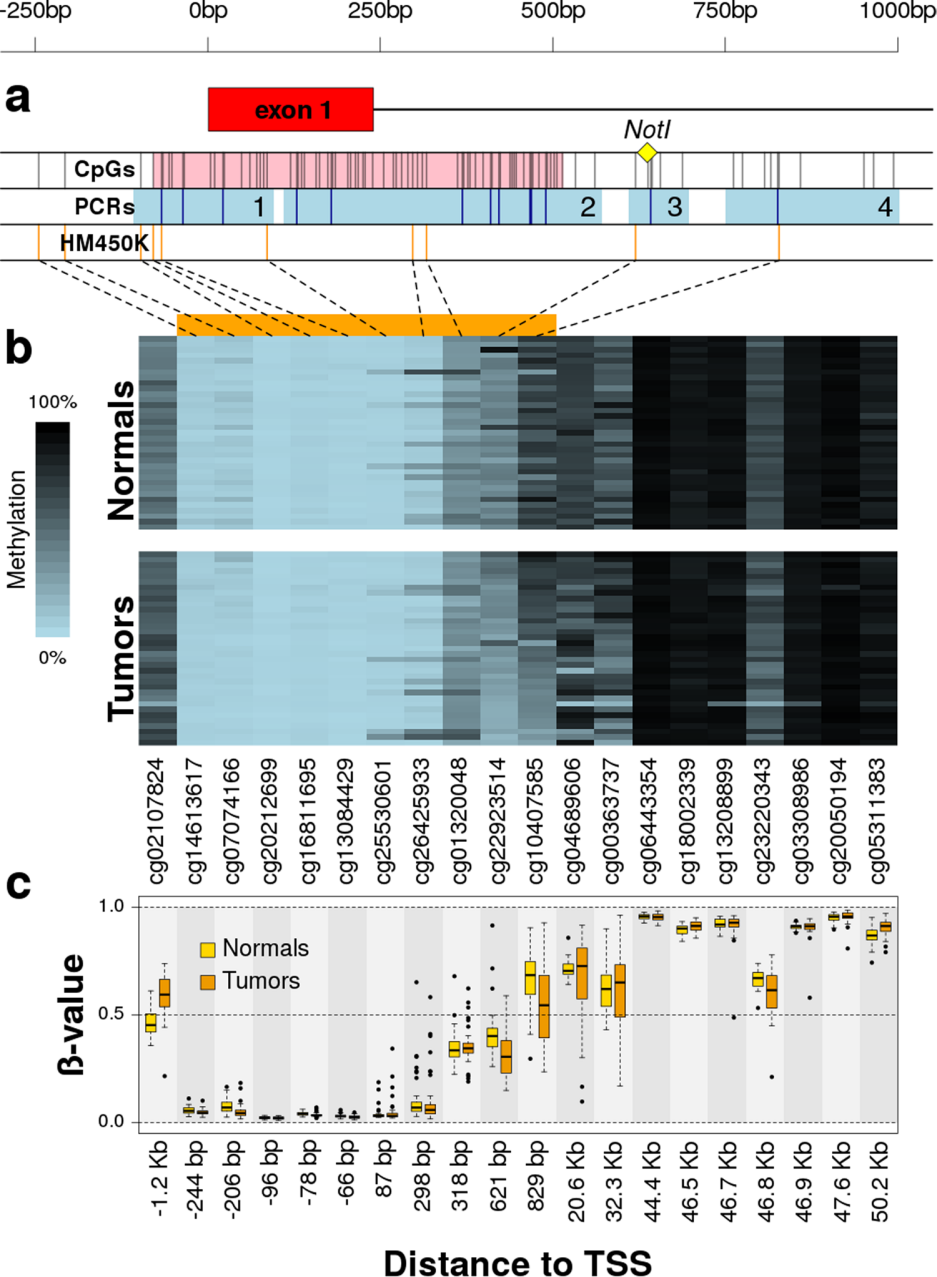
Methylation of *VWA2* locus in 35 pairs of matched normal and tumor tissues from CRC patients. Panel a: Scheme of the 5′-associated region of *VWA2*. Symbols and tracks like in Fig. 1. Panel b: methylation heatmap of the Illumina HM450K array probes within the *VWA2* locus in normal and tumor tissues from 35 CRC patients. Methylation is represented in a blue to black gradient. The horizontal orange bar on top of the heatmap indicates those probes within the 5′-associated region shown in panel a. Patients are ordered in the vertical axis according to the average methylation of probes cg22923514 and cg10407585 in tumors. Probes are ordered in the horizontal axis according to their location in the genome. Panel c: boxplot of the ß-values of these probes in normal (yellow) and tumor (orange) tissues from the 35 patients shown in panel b. The distance to the transcriptional start site (TSS) of every probe is indicated in the x-axis.

We profiled the methylation of this region in 35 primary tumors and their matched normal samples using Illumina HM450K methylation arrays. These arrays contain 22 probes within the *VWA2* locus ±1.5Kb, eight of them overlapping the region previously studied by PCR (Fig. 2a). Two probes were excluded during the computational preprocessing step: probe cg24882714 because it overlaps with SNP rs56041166 (MAF = 8% in European populations), and probe cg24242022 which was removed by the Greedycut algorithm that controls the reliability of the methylation call^16^. For the remaining 20 probes, we calculated the average methylation difference in ß-units between tumor and normal samples (∆ß, Supplementary Table ST3). In agreement with the bisulfite sequencing and COBRA results, the Illumina HM450K analysis showed very small methylation differences between normal and tumor tissues (∆ß < 0.02) within the CGI that overlaps the TSS and first exon, albeit some of them reached statistical significance (*P* < 0.01 paired t-test). Probes cg22923514 (region 3, ∆ß = −0.096, *P* = 1.97 × 10^−4^) and cg10407585 (region 4, ∆ß = −0.129, *P* = 4.16 × 10^−3^) were clearly hypomethylated in tumors compared to normals (Fig. 2b,c), with a strong correlation between them (r = 0.64, *P* = 2.97 × 10^−5^). That correlation, together with their physical proximity (~200 bp), suggested that they signal the same somatic hypomethylation event taking place at the south shore of the *VWA2* CGI. Probe cg02107824, located 1.2Kb upstream of the TSS and outside of the region previously analyzed by PCR, exhibited somatic hypermethylation (∆ß = 0.123, *P* = 1.09 × 10^−7^). We also found statistically significant somatic methylation changes in another three probes (cg18002339, cg23220343 and cg05311383) located much farther from the TSS, in the +46 to +50 Kb downstream region.

Somatic methylation changes of the *VWA2* locus were additionally validated using the publicly available data from colon (COAD) and rectal (READ) cancer datasets from The Cancer Genome Atlas (TCGA)^17^. In total, 352 CRC samples and 45 normal colonic mucosa samples with methylation information were available, derived from 350 CRC patients (tumor samples from two patients were analyzed in duplicate, from two separate vials each). The TCGA methylation analysis pipeline excluded cg20212699 in all samples, and cg02107824 was excluded in COAD samples (Supplementary Fig. S3). This larger dataset confirmed our original observations: very small somatic methylation changes in the *VWA2* CpG island except for the probes in the region 3 (cg22923514) and 4 (cg10407585), that were significantly hypomethylated in tumors (*P* = 5.5 × 10^−6^ and *P* = 4.4 × 10^−6^, respectively). Exactly as previously observed in our tumor collection, the methylation levels reported by these two probes in the TCGA data exhibited a strong intercorrelation (r = 0.68, *P* = 2.7 × 10^−48^), once again suggesting that they both signal the same demethylation event. The probe cg02107824, located 1.2Kb upstream of the TSS, was clearly hypermethylated in TCGA tumors, also in agreement with our previous results. Other probes located in the gene body, over 20Kb downstream of the TSS, exhibited also statistically significant somatic methylation changes (Supplementary Table ST3).

### *VWA2* undergoes epigenetic remodeling in CRC

Somatic changes in histone marks in the *VWA2* region were analyzed by chromatin immunoprecipitation using formalin-fixed paraffin-embedded (FFPE) normal and tumor samples from three CRC patients previously analyzed by Illumina methylation arrays. Case 719 did not exhibit somatic changes in DNA methylation in the *VWA2* CGI south shore (probes cg22923514 and cg10407585), while cases 726 and 727 showed somatic hypomethylation of this region.

**Figure 3:**
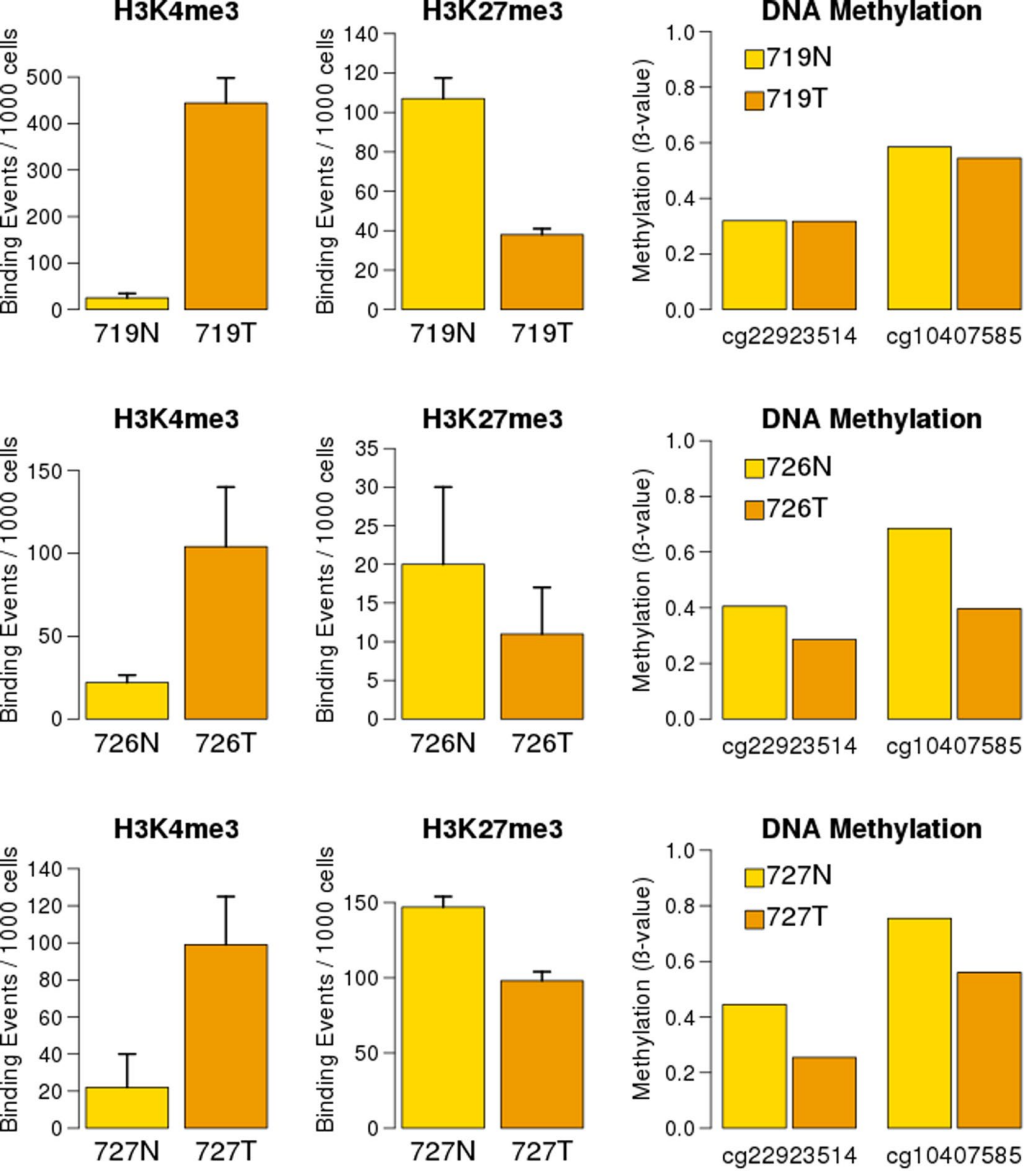
Histone marks and DNA methylation in the *VWA2* promoter region of normal (N, in yellow) and tumor (T, in orange) tissues from CRC patients 719, 726 and 727. Two different histone modifications, i.e. H3K4me3 and H3K27me3, were analyzed by FFPE ChIP-Seq. These three cases exhibited a significant increase in the active-chromatin mark H3K4me3 (left) and a significant decrease in the repressive-chromatin mark H3K27me3 (middle). Methylation values of Illumina HM450K array probes cg22923514 and cg10407585, located in the *VWA2* CGI south shore, are shown (right). Case 719 did not exhibit clear changes in methylation. Cases 726 and 727 exhibited somatic hypomethylation at both probes.

The results revealed a significant increase of histone mark H3K4me3 and decrease of H3K27me3 in the 5′ region of *VWA2*, consistent with an epigenetic remodeling from transcriptionally inactive to transcriptionally active chromatin (Fig. 3). Notably, epigenetic remodeling was observed in the three studied cases, including case 719 that was not somatically hypomethylated according to the Illumina HM450K array analysis.

### Hypomethylation of *VWA2* correlates with mRNA expression in primary cancers

The TCGA COAD and READ datasets were employed to study the association between *VWA2* methylation and transcriptional expression. Transcriptional expression (RNAseq) data from 594 CRC samples was obtained from the release 20 of the International Cancer Genome Consortium (ICGC). These samples derived from 581 patients (in thirteen of them, 2 different sections of the tumor were analyzed). For the *VWA2* methylation-expression association analysis, we employed 348 samples that have both *VWA2* methylation and expression information, derived from 346 patients.

Eight methylation probes within the *VWA2 locus* exhibited statistically significant association with *VWA2* expression (*P* < 0.05 after multi-hypothesis testing correction, Supplementary Fig. S4). Three of them (cg13208899, cg03308986 and cg05311383), located 46.7 to 50.2 kb downstream of the TSS, exhibited positive association with mRNA expression, i.e. the higher the DNA methylation the higher the mRNA expression, despite the fact that these probes were mostly methylated (ß-value > 0.8) in the majority of the tumors and in all normal samples (Figs 2 and S3). Two CGI south shore probes exhibited negative association with expression, i.e. lower levels of methylation correlated with higher levels of expression. These probes were cg22923514 (region 3, 0.6 kb downstream of the TSS, r = −0.27, r^2^ = 0.08, *P* = 3 × 10^−6^) and cg10407585 (region 4, 0.8 kb downstream of the TSS, r = −0.5, r^2^ = 0.25, *P* = 1.6 × 10^−22^). Of these two probes, the probe in region 4 (cg10407585) was considerably more sensitive to detect somatic hypomethylation and also exhibited a stronger correlation with expression (Supplementary Fig. S4).

**Figure 4:**
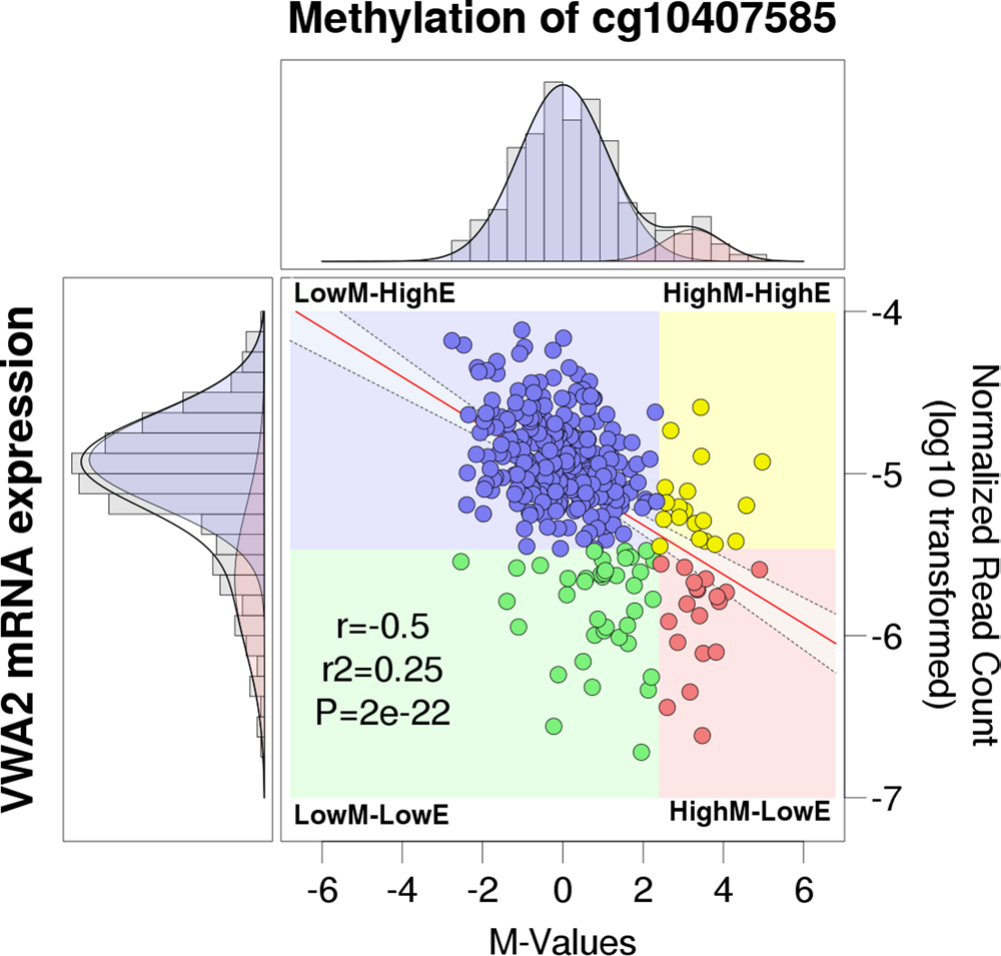
Correlation between methylation of probe cg10407585 and *VWA2* mRNA expression in 348 CRC tumors from the TCGA. In the central dot plot, methylation is represented as M-values in the x-axis. M-values of -2 and 2 correspond to ß-values of 0.2 and 0.8, commonly considered thresholds for demethylation and full methylation, respectively. *VWA2* mRNA expression is represented in the y-axis, as the log10 transformation of the normalized read counts. The regression line is in red, with the 95% confidence interval of the slope represented by the shaded area between the dashed lines. The Pearson’s product-moment correlation coefficient (r), the coefficient of determination (r2) and the *P*-value (P) after correction for multi-hypothesis testing are indicated. In the density graphs of the M-values (top) and log10-transformed normalized read counts (left), grey bars represent the histograms of the observed distributions. The solid black lines represent the expected density of fitted Gaussian mixture models (see Supplementary Fig. S5). For methylation (top), the model yielded a large group (89%, shaded in blue) with low to intermediate levels of methylation, and a smaller group (11%, shaded in pink) with high levels of methylation. For expression, the model yielded a large group (78%, shaded in blue) with higher expression and a small group (21%, shaded in pink) with lower expression. Combining both binary classifications, cases were divided into low methylation – high expression (LowM-HighE, 77.87%, blue dots), high methylation – high expression (HighM-HighE, 5.46%, yellow dots), low methylation – low expression (LowM-LowE, 11.21%, green dots) and high methylation – low expression (HighM-LowE, 5.46%, pink dots).

Both cg10407585 methylation and *VWA2* expression values in primary CRCs followed bimodal distributions (Supplementary Fig. S5), suggestive of the existence of two distinctive groups of tumors. Using the posterior probabilities of the binomial distribution models, tumors were classified into four groups: low methylation and high expression (n = 271, 77.9%, LowM-HighE), low methylation and low expression (n = 39, 11.2%, LowM-LowE), high methylation and high expression (n = 19, 5.5%, HighM-HighE) and high methylation and low expression (n = 19, 5.5%, HighM-LowE). The association between methylation-based classification and expression-based classification was very high (OR = 6.9, CI = 3.2–15.1, *P* = 3.4 × 19^−7^, Fisher’s exact test) (Fig. 4).

No statistically significant differences in clinicopathological characteristics (patient age, gender, race, weight, tumor location, mucinous phenotype and existence of synchronous cancers) were found between TCGA tumors with low vs high cg10407585 methylation, or with low vs high *VWA2* expression (Supplementary Table ST4).

### Treatment with AZA-2-deoxycytidine reactivates expression of *VWA2* in cell lines

**Figure 5:**
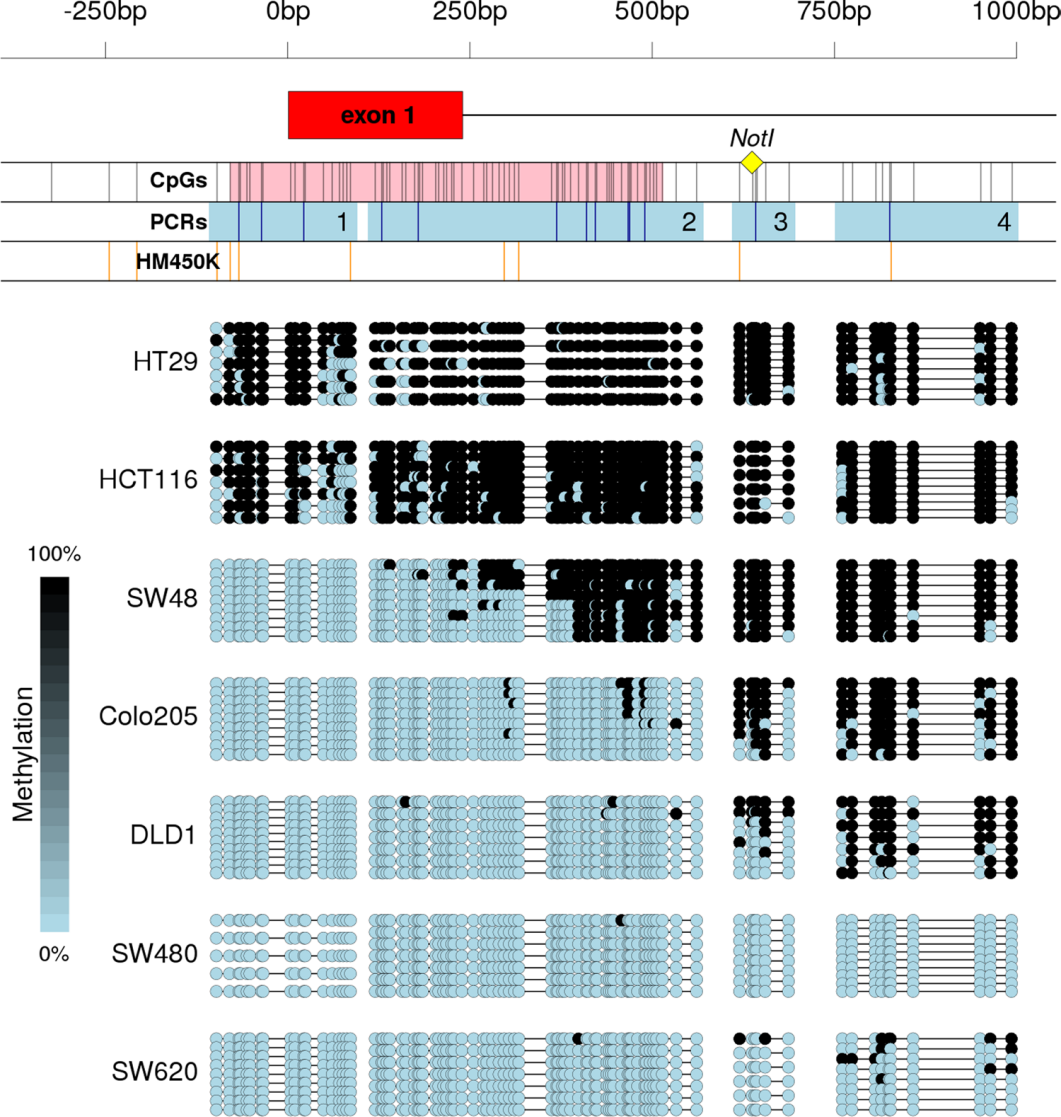
Methylation of the 5′-associated region of *VWA2* in CRC cell lines. On top, a scheme of the region. Symbols and tracks identical to those in Fig. 1. At the bottom, bisulfite sequencing results. Analyses were performed in 4 separate PCRs. Every line represents and individually sequenced clone. Dots represent unmethylated (blue) or methylated (black) CpG sites. Primary cancer-derived cell lines are organized from higher (HT29) to lower (SW480) methylation levels. The lymph node-derived cell line SW620 is shown at the bottom.

We analyzed *VWA2* methylation by bisulfite sequencing in 7 colorectal cancer cell lines (Fig. 5). Paradoxically, in contrast with the initial observation where hypermethylation of the *VWA2* CGI was never detected in primary tumors, this region was heavily methylated in 2 of the CRC cell lines, namely HT29 and HCT116. In SW48, the CGI was partially methylated, showing a similar pattern to that found in normal colonic tissues. In DLD1, hypomethylation was clearly detected in region 3. Finally, in SW480 and SW620, originated from a primary tumor and a lymph node metastasis from the same patient, the four analyzed regions were essentially hypomethylated.

**Figure 6:**
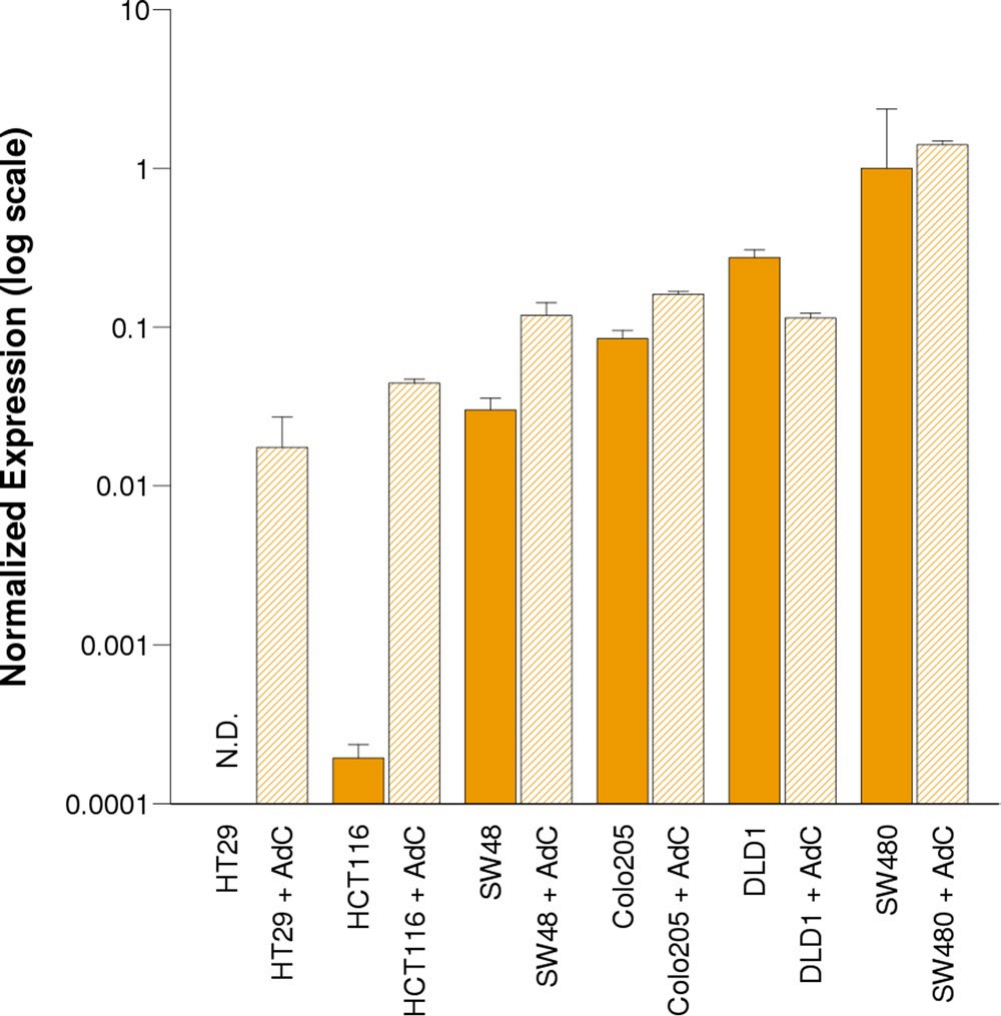
*VWA2* trancriptional expression measured by qPCR in cell lines derived from primary CRC. Cells are ordered according to the methylation level in the VWA2 5′ region (Fig. 5), from more methylated (HT29) to less methylated (SW480). mRNA levels were normalized against the highest expressing cell line (SW480) using *GAPDH* and *TPT1* as reference housekeeping genes. Bars indicate *VWA2* mRNA levels after 48 h of treatment with DMSO (solid bars) or DMSO + 2-deoxy-AZA-cytidine 1 µM (AdC, dashed bars). Experiments were performed in triplicate. Error bars indicate the standard error. N.D.: mRNA not detected after 40 cycles of PCR.

The association between *VWA2* DNA methylation and transcriptional expression was studied only in primary cancer derived cell lines, after excluding the lymph-node metastatic cell line SW620 (Fig. 6). The highest-expressing cell line was SW480, which also exhibited the lowest levels of methylation. Expression gradually declined with increasing methylation in DLD1, Colo205 and SW48, and reached the lowest values in the highly methylated lines HCT116 and HT29 that exhibited less than one thousand and ten thousand times lower levels than SW480, respectively, demonstrating a strong negative correlation between DNA methylation and gene expression (Spearman’s rank correlation rho = 1, *P* = 0.003). Upon 48 h treatment with 1 µM of the DNMTs inhibitor 5-AZA-2-deoxycytidine (AdC), *VWA2* expression was greatly induced in HCT116 and HT29, and to a lower extent in SW48 and Colo205 (Fig. 6).

### Up-regulation of *VWA2* associates with increased expression of WNT/Myc target genes

**Figure 7:**
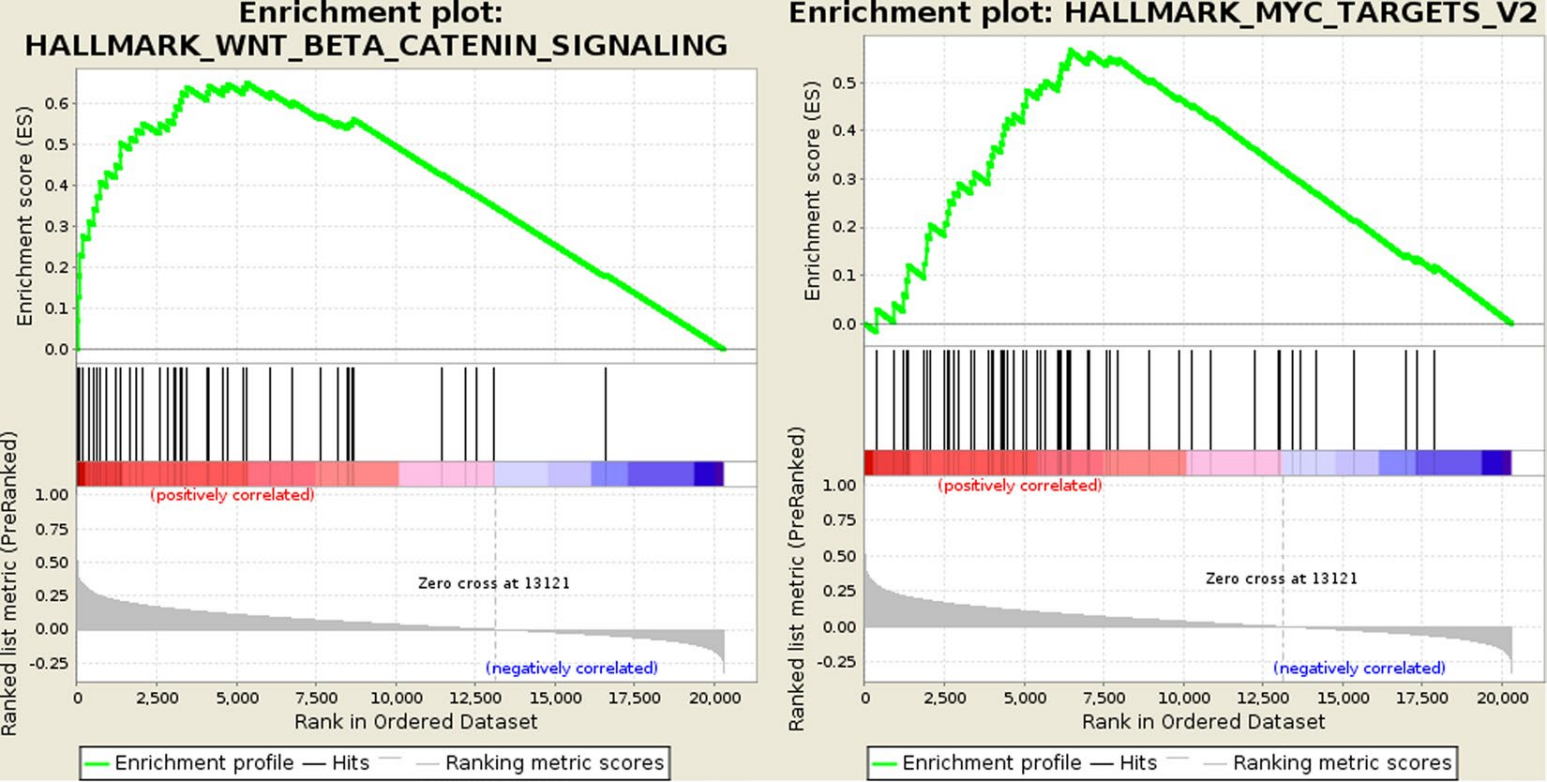
Gene set enrichment analysis performed with GSEA on 20501 genes list sorted according to the correlation between their expression level and *VWA2* expression in 594 CRC cancers from the TCGA. The two gen sets with strongest association were HALLMARK_WNT_BETA_CATENIN_SIGNALING (MutSigDB M5895, genes up-regulated by activation of WNT signaling through accumulation of beta catenin CTNNB1, left) and HALLMARK_MYC_TARGETS_V2 (MutSigDB M5928, A subgroup of genes regulated by MYC - version 2, right). A complete list of results is available in Supplementary Table ST6.

Using the RNAseq data from the COAD and READ datasets from the TCGA (20501 genes in 594 colorectal cancers), we calculated the correlation between *VWA2* transcriptional levels and that of the remaining 20500 genes (Supplementary Table ST5). gene set enrichment analysis (GSEA) revealed that *VWA2* expression correlated with several gene sets from the hallmark collection of the molecular signatures database (MSigDB, Supplementary Table ST6)^18,19^, most notably genes activated by WNT signaling (MSigDB gene set M5895) and Myc-target genes (MSigDB gene set M5928) (Fig. 7).

Moreover, somatic up-regulation of *VWA2* was not accompanied by up-regulation of neighboring genes. Of the 55 genes within the 10 Mb region surrounding *VWA2 locus*, *VWA2* exhibited the highest up-regulation in tumors (16.6 fold change compared with normal tissues, Supplementary Fig. S6). Two other genes, namely *RPL13AP6* and *HABP2*, were also up-regulated but to a lower extent (4.1 and 8.9 fold change, respectively). The vast majority of the genes in that region did not exhibit significant changes (n = 38) or were down-regulated in tumors (n = 14). This observation suggested that *VWA2* up-regulation is a targeted phenomenon rather than a co-lateral effect of copy number amplification targeting a neighboring gene.

In view of these results, and considering the relatively high correlation between *VWA2* and *MYC* expression (r = 0.297, *P* = 1.4 × 10^−7^, among the top 1.3% correlated genes), we scanned the DNA sequence of *VWA2* for putative Myc-binding motifs that could suggest a direct regulation mechanism^20,21^. Albeit *VWA2* was not included among the MSigDB hallmark myc targets gene sets M5926 or M5928, nor reported among the 257 genes of the high Myc-affinity group in a comprehensive ChIP-Seq scan performed in 6 different human cell lines^22^, we found a perfect Myc binding motif (5′-tccCACGTGgca-3′, in uppercase the consensus E-box hexamer) located 120 bp downstream of the *VWA2* TSS, within its promoter-associated CpG island. This motif complies with the typical characteristics of high-affinity E-boxes, i.e. perfect hexamer within an unmethylated CpG-rich sequence.

At the present time, ENCODE has generated c-myc ChIP-seq data for 9 different cell lines (K562, MCF-7, HeLa-S3, MCF10A, A549, GM12878, HepG2, NB4 and H1-hESC, see methods), albeit none of the 6 CRC lines included in our study. This data revealed a significant enrichment of c-myc at the E-box containing *VWA2* DNA region in two cancer cell lines, MCF-7 (breast cancer) and HepG2 (hepatocellular carcinoma) and in the embryonic stem cells H1-hESC (Supplementary Fig. S7), supporting that c-myc can directly bind this DNA region in cells where *MYC* is highly expressed^23^.

**Figure 8:**
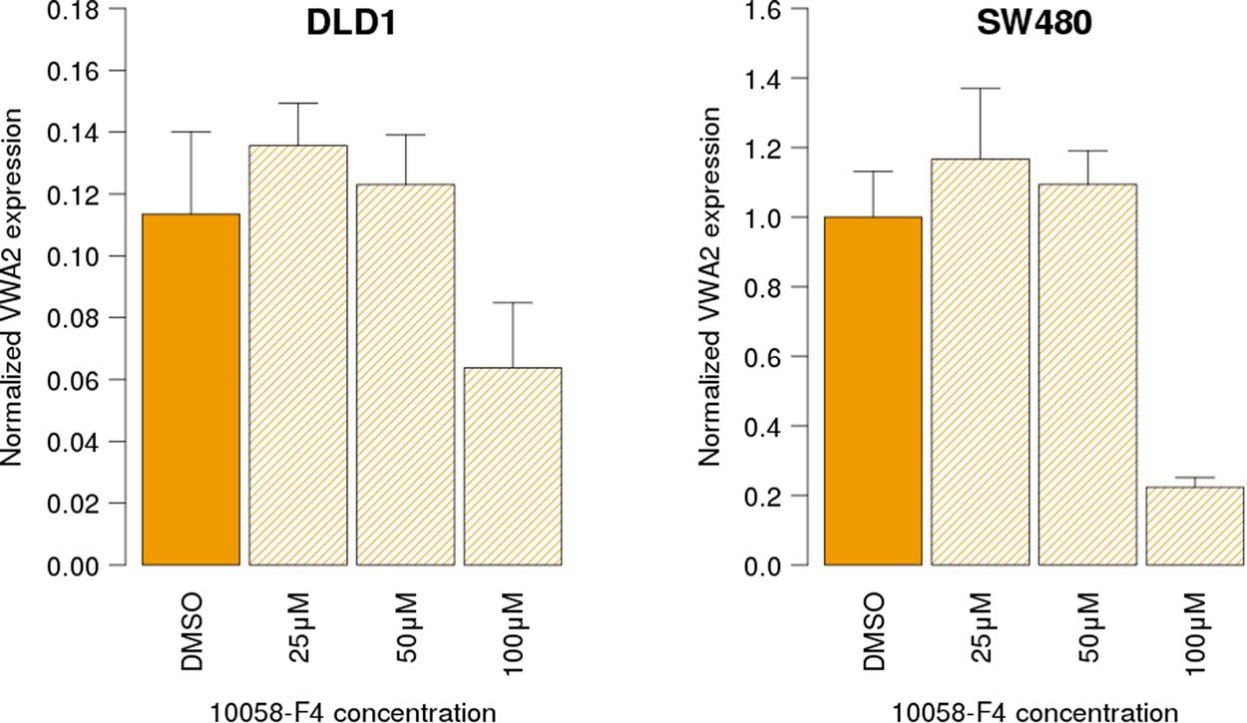
*VWA2* transcriptional levels in DLD1 (left) and SW480 (right) CRC cell lines, after 48 h treatment with the c-myc inhibitor 10058-F4 at different concentrations. Solid bars indicate control cells treated with the vehicle (DMSO). Dashed bars indicated the cells treated with vehicle + 25 µM, 50 µM and 100 µM 10058-F4. mRNA levels were normalized against the highest expressing cell line (SW480) using *GAPDH* and *TPT1* as reference housekeeping genes. Experiments were performed in duplicate. Error bars indicate the standard error.

Given the accumulation of indirect evidence suggestion a functional role of c-myc on the expression of *VWA2*, we tested this association by treating DLD1 and SW480 cell lines (both myc+ and exhibiting the highest expression level of *VWA2*, Fig. 6) with the c-myc inhibitor 10058-F4 for 48 h at three different concentrations^24,25^. The results revealed a significant reduction of *VWA2* expression upon treatment with 100 µM 10058-F4 (Fig. 8), further supporting a functional link between c-myc and *VWA2* up-regulation.

### Expression of *VWA2* in other cancer types

**Figure 9:**
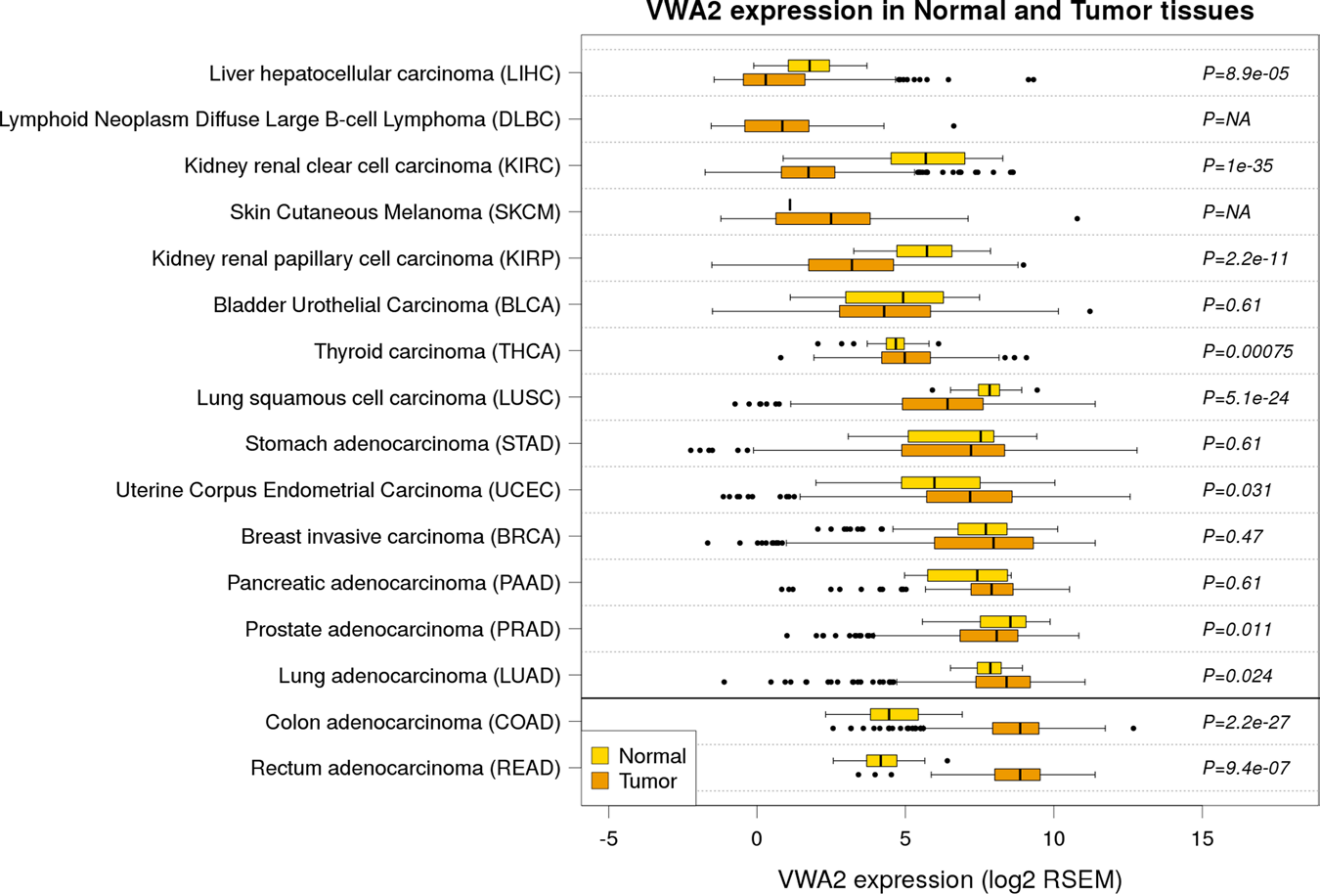
*VWA2* transcriptional levels in normal (yellow) and tumor (orange) tissues in the most common types of cancer from the TCGA datasets. *P-*values were calculated by Student’s t-test. Not enough data from normal DLBC and SKCM tissues was available to perform the statistical test (P = NA).

The analysis of *VWA2* expression in normal and tumor tissues from 34 different cancer cohorts with RNAseq data available at the TCGA revealed that somatic over-expression of this gene is particularly strong and frequent in colon and rectal cancers. Other types of cancer exhibited the opposite trend, i.e. somatic downregulation of *VWA2*, in particular kidney cancers, lung squamous cell carcinoma and head and neck squamous cell carcinomas (Supplementary Table ST7 and Supplementary Fig. S8). Among the 15 most common cancers (as defined by the National Cancer Institute of the NIH, https://www.cancer.gov/types/common-cancers)^26^, colorectal cancer is clearly the one that exhibits the most dramatic somatic up-regulation of *VWA2*, both in frequency and magnitude (Fig. 9).

## Discussion

*VWA2* had been found to be over-expressed in the majority of colorectal tumors, including adenomas and carcinomas^10,11^. This gene has a CpG island encompassing its canonical transcriptional start site and its first non-coding exon. Based on the existence of a promoter-associated CGI, *VWA2* would be susceptible to epigenetic regulation by DNA methylation. Our results show that this CpG island, however, did not undergo dramatic somatic methylation changes in primary CRCs, remaining mostly unmethylated in both normal and tumor samples (Fig. 1). However, the CpG island shore downstream of the first exon underwent clear somatic changes in methylation (Figs 1, 2 and Supplementary Fig. S2). DNA hypomethylation associated with an increase of H3K4me3 and a decrease of H3K27me3, consistent with transcriptional activation (Fig. 3).

The analyses both in our tumor collection (Fig. 2) and in the TCGA collection (Fig. S3) using Illumina HM450K arrays demonstrated that the 5′ region of the *VWA2* CGI underwent negligible changes in methylation in CRC primary tumors, while the 3′ region of the CGI and, particularly, the south shore interrogated by probes cg22923514 (region 3) and cg10407585 (region 4) exhibited significant hypomethylation that correlates with transcriptional up-regulation (Fig. S4).

Our initial MS-AFLP analyses yielded an incidence of somatic hypomethylation much lower (17%) than the reported frequency of over-expression in primary cancers (80%)^10^. This apparent discrepancy was likely due to the very strict criterion that we employed to classify *VWA2* hypomethylation using MS-AFLP (see methods), leading to a high probability of false negative error. Bisulfite sequencing revealed that demethylation of the *VWA2* CGI shore was a gradual phenomenon spreading from the CGI downstream towards the PCR regions 3 and 4. The *Not*I site interrogated by MS-AFLP was located in the PCR region 3, where the differences between normal and tumor tissues were not as dramatic as in the downstream PCR region 4. That region is interrogated by the Illumina HM450K probe cg10407585, that showed very high sensitivity to detect somatic changes in methylation (Fig. 2 and Supplementary S3) and also the strongest correlation with *VWA2* expression levels (Fig. 4 and Supplementary S4). Both our tumor collection and the TCGA dataset revealed the existence of two distinct groups of tumors according to the methylation levels of the probe cg10407585. The larger group, comprising around 80% of the tumors, exhibited low to intermediate methylation values (Fig. 4). This group of tumors was largely overlapping with the group of tumors with higher *VWA2* expression. Classifying the tumors according to both methylation and expression revealed that the vast majority of the tumors (271/348, 78%) belong to the low-methylation and high-expression group. That analysis also revealed a small percentage of tumors (5.5%) that exhibited over-expression of *VWA2* but not somatic DNA hypomethylation. Conversely, a small percentage of tumors (11%) exhibited somatic DNA hypomethylation but not transcriptional activation. This suggests that DNA hypomethylation and transcriptional activation, albeit strongly inter-related, might also occur independently. In line with this observation, our FFPE ChIP-Seq experiments showed that case 719 exhibited epigenetic remodeling consistent with transcriptional reactivation, but not somatic DNA demethylation (Fig. 3).

When we first identified *VWA2* somatic hypomethylation using MS-AFLP, we found that it associated with patient age (Table 1). However, the methylation levels of the probe cg10407585 both in our dataset and the TCGA COAD + READ datasets did not associate with patient age (Supplementary Table ST4). This discrepancy might be explained, once again, by the extremely strict criterion applied to score the MS-AFLP results. The lack of association with age suggests that *VWA2* hypomethylation is a targeted event rather than just a consequence of the age-related genomewide hypomethylation that takes place in essentially all dividing tissues^13,27^.

Expression of *VWA2* in primary tumors correlated with expression c-Myc, and with that of Myc target genes, suggesting that *VWA2* might be part of an oncogenic transcriptional program driven by c-Myc^28–30^. In line with this observation, we found a perfect E-box motif 120 bp downstream of the *VWA2* TSS within its promoter-associated CpG island, suggesting that *VWA2* could be an unreported direct target of Myc in CRC. The data from the ENCODE project revealed c-myc enrichment at this region in several MYC-expressing cell lines (Supplementary Fig. S7), with the enrichment peaks centered on the E-box motif located in the first exon of *VWA2*. In addition, treatment with the c-myc inhibitor 10058-F4 reduced the transcriptional levels of *VWA2* in DLD1 and SW480 cells (Fig. 8). Both cell lines exhibit a strong up-regulation of *MYC*, which might explain the requirement of a relatively high concentration (100 µM) of 10058-F4 to induce transcriptional down-regulation of *VWA2*.

Paradoxically, HT29 and HCT116 cell lines exhibited strong hypermethylation and transcriptional silencing of *VWA2* (Figs 5 and 6), essentially the opposite phenomenon to that observed in primary cancers. None of the primary tumors in our collection (n = 35) or in the TCGA collection (n = 352) exhibited hypermethylation of the *VWA2* CGI. This apparently contradictory finding suggests that while *VWA2* might play a pro-tumoral role in oncogenesis or during tumor progression, its function would not be required and perhaps even subjected to negative selection during *in-vitro* cell culturing, thereby leading to culture-induced epigenetic silencing. *De novo* methylation of CpG islands upon adaptation to cell culturing conditions is a long observed and common phenomenon^31^, calling for caution before extrapolating findings from cell lines to primary cancers. If the pro-tumoral effect of *VWA2* is associated with the activation of an oncogenic transcriptional program driven by Myc, as suggested by the co-regulation analysis, it can be speculated that over-expression of *VWA2* would become irrelevant for cancer cells once they become adapted to *in vitro* culture conditions. A previous study exploring epigenetic changes induced by cell culture in mouse embryonic fibroblasts (MEFs) revealed global loss of 5-hydroxymethylcytosine (5hmC, an intermediate of methyl-cytosine demethylation by an active mechanism) upon adaptation to culture conditions^32^. Notably, *Vwa2* was one of the genes found to undergo loss of 5hmC. Whether this observation in mouse MEFs would be also applicable to human colorectal cancer cells is uncertain, considering the different nature of these cells, but it opens the interesting question regarding the mechanism (active or passive) responsible for the observed hypomethylation of *VWA2*^33^.

AdC-induced DNA demethylation significantly increased *VWA2* transcription in all studied CRC cancer cell lines except DLD1 and SW480 (Fig. 6), both with mostly hypomethylated region 3 where the *Not*I site is located, suggesting that hypomethylation of this region facilitates *VWA2* transcription. Due to the genomewide effect of AdC, we cannot determine whether the pharmacological demethylation exerts a direct effect on *VWA2* transcription, i.e. through demethylation of the *VWA2* locus, relaxing the chromatin structure and facilitating the access of the transcriptional machinery, or indirect, i.e. through activation of a transcriptional factor in *trans*.

The pharmacological induction and repression experiments shown in Figs 6 and 8 were conducted using a QPCR primer combination targeting exons 8–10. Notably, a regulated ncRNA variant of *VWA2* lacking exons 1 to 5 was originally reported in adult mouse kidney^2^. We confirmed that the up-regulated *VWA2* mRNA also contained the initial coding exons, by analyzing both the AdC-induced transcriptional activation in HT29 and HCT116, and the down-regulation induced by 10058-F4 in SW480, using two additional sets of primers targeting exons 2–4 (Supplementary Fig. S9).

In conclusion, our results revealed the link between somatic DNA hypomethylation of the *VWA2* CGI shore and the aberrant over-expression of this gene in primary colorectal cancers, exemplifying how epigenetic alterations taking place in the proximity of a promoter-associated CpG island may correlate with gene transcription even when the CpG island itself remains essentially unaltered^34^. Our results also unveiled an apparently paradoxical observation: *VWA2* becomes over-expressed in primary CRCs but epigenetically repressed in some CRC cell lines, suggesting that the putative pro-tumoral effect of *VWA2* is not required (and perhaps even detrimental) for CRC cells growing in culture. In addition, we found compelling evidence that *VWA2* is one of the targets of a transcriptional program regulated by Myc. Finally, our study revealed that over-expression of *VWA2* is virtually exclusive of colorectal cancers (Fig. 8) which, together with fact that it has been also found over-expressed in adenomas^10^, reinforces its potential as a highly sensitive and specific biomarker for early detection of CRC^11,35^.

## Methods

### Tumor samples and colorectal cancer cell lines

Eighty-one primary colon cancers with matched normal tissue were obtained as frozen specimens and FFPE samples from the Cooperative Human Tissue Network (CHTN, https://www.chtn.org/). The CHTN is a unique resource supported by the National Cancer Institute of the USA, providing human tissues and fluids from routine procedures to investigators who utilize human biospecimens in their research. Informed consent was obtained and managed by the CHTN at the time of sample collection, following US regulations and the guidelines of the Declaration of Helsinki^36^. Our research group has been collecting cancer samples from the CHTN for over 20 years, when based at the Sanford-Burnham-Prebys Medical Discovery Institute, formerly Sanford-Burnham Medical Research Institute (SBMRI). Approval to collect samples, extract DNA and perform analyses was obtained at the time from the Institutional Review Board (IRB) of the SBMRI. Part of the extracted DNA collection has been transferred to our laboratory at the IGTP, Barcelona, Spain. Additional approval was obtained from the IRB of the IGTP to further analyze the DNA samples employed in this study. Seven colorectal cancer cell lines were obtained from the ATCC: HT29 (ATCC HTB-38), HCT116 (ATCC CCL-247), SW48 (ATCC CCL-231), Colo205 (ATCC CCL-222), DLD1 (ATCC CCL-221), SW480 (ATCC CCL-228), SW620 (ATCC CCL-227).

### Cell culture conditions

Cells were cultured in DMEM-F12 (Life Technologies) supplemented with fetal bovine serum 10% (*v/v*), antibiotics, and antimycotics on 100 mm culture dishes in a 37 °C incubator with 5% CO2. Unless otherwise indicated, cells were grown until reaching 80–90% confluency before collection. To induce DNA demethylation, 5-aza-2′-deoxycytidine (5AdC, Sigma-Aldrich, CatNo. A3656) was added to the culture media at a final concentration of 1 µM, from a 500 µM working solution. Myc inhibitor 10058-F4 was purchased from Sigma-Aldrich (CatNo F3680). Stocks were prepared at 2.5 mM, 5 mM and 10 mM in DMSO. Final concentration was 2.5 µM, 5 µM and 10 µM. Controls were subjected to the same incubation conditions but adding only the vehicle at the appropriate volume.

### Methylation-sensitive amplified fragment length polymorphism (MS-AFLP)

MS-AFLP was performed as described previously. Briefly, 1 µg of genomic DNA was digested overnight with 5 units of methylation-sensitive *Not*I (Roche, Indianapolis, IN) and 2 units of methylation-insensitive *Mse*I (NE Biolabs, Beverly, MA) at 37 °C. Two pairs of oligonucleotides were annealed overnight at 37 °C to generate *Not*I and *Mse*I specific adaptors. The digested DNA was ligated to 1.25 ml each of 5 pmol/ml *Not*I and 50 pmol/ml *Mse*I adaptor using 1 unit of T4 DNA ligase (Roche) overnight at 16 °C. A primer complementary to the NotI adaptor (*Not*I primer) was labeled at the 5′ end using ^32^P-γ-ATP and T4 polynucleotide kinase (Promega, Madison, WI). The adaptor-ligated template DNA was PCR amplified using ^32^P-labeled *Not*I and *Mse*I-C primers. A total of 20 µl of PCR mixture consisted of 6 ng of 32P-labeled *Not*I primer, 30 ng of *Mse*I-C primer, 0.4 mM dNTP, and 1 unit of AmpliTaq DNA polymerase (Perkin-Elmer, Foster City, CA). Two different amounts of template DNA (2 ng and 4 ng) were used to confirm the reproducibility of the results. The PCR started at 72 °C for 30 s and 94 °C for 30 s, then was followed by 35 cycles of 94 °C for 30 s, 52 °C for 30 s, and 72 °C for 2 min. The final extension was performed for 10 min at 72 °C. Each PCR sample was electrophoresed on a denaturing gel (Sequagel-6, National Diagnostics, Atlanta, GA) after heat denaturing. The gel was dried and exposed to an X-ray film. Primer sequences are detailed in Supplementary Table ST1.

### Scoring of methylation alterations in tumors by MS-AFLP

Independent DNA fingerprints were run on acrylamide gels in duplicate or triplicate. Scoring of quantitative changes between normal and tumor DNA was made by visual inspection independently by three researchers, as described previously^14,37^. Somatic alterations were scored on bands with consistent pattern among normal tissues, with clear changes in intensity in tumor samples and called by at least two of the three researchers.

### COBRA and bisulfite genomic sequencing

Genomic DNA was treated with sodium bisulfite using EZ DNA Methylation Kit (Zymo Research, Orange, CA). Four different semi-nested PCRs were designed to cover a total of 1.2 kB, including the CpG island upstream of Exon 1 and the location of the band previously identified by MS-AFLP. Primers are described on Supplementary Table ST1. For COBRA, after amplification, the PCR products were subjected to digestion with the restriction endonuclease *Bst*UI (New England Biolabs, Ipswich, MA) or its isoschizomer Bsh12361 (Thermofisher Scientific), and subsequently resolved on agarose gels. For bisulfite genomic sequencing the PCR products were cloned into StrataClone SoloPack Competent Cells using StrataClone PCR Cloning Kit (Agilent, Life Technologies, CA). Cells were cultured on LB plates containing Kanamycin 30 µg/ml and XGal 20 µg/ml. Plasmids from white positive colonies were isolated using Nucleospin Plasmid EasyPure (Macherey-Nagel GmbH & Co. KG, Dueren, Germany). Sequencing of the cloned PCR products was performed by GATC BiotechAG (Germany) using T3 universal primer.

### Methylation arrays

Genomewide methylation was assayed using Illumina HM450K Methylation arrays, and processed using RnBeads software^16^. In most figures, methylation was generally represented in the more intuitive ß-value units, which range between 0 (unmethylated cytosine) and 1 (methylated cytosine), but statistical analyses were performed on the M-values since they exhibit lower heterocedasticity than ß-values^38^.

### Chromatin immunoprecipitation

Chromatin was prepared from using ChIP-IT^®^ FFPE from Active Motif and subsequently subjected to immunoprecipitation with H3K4me3 (active transcription mark) and H3K27me3 (inhibitory mark) antibodies, following the manufacturer protocol. After reverse cross-linking, immunoprecipitated DNA was used to prepare genomic libraries that were sequenced in an Illumina NGS sequencer following standard procedures.

### Gene expression analyses

To analyze *VWA2* mRNA levels, total RNA was extracted using Maxwell® 16 LEV simplyRNA Purification Kit (Promega) and the Maxwell® 16 Instrument MX3031 (Promega, CatN° AS2000) following the supplier protocol. The purified RNA was used as template to synthesize cDNA using Superscript-II reverse transcriptase (Invitrogen, Life Technologies) with random hexamers for priming. We designed three primer pairs to analyze *VWA2* expression (Supplementary Table ST1), amplifying exons 8–10 (PB66 + PB67, 150 bp amplicon size) or exons 2–4 (PB271 + PB272, 203 bp amplicon size, and PB273 + PB274, 198 bp amplicon size). The results shown in Figs 6 and 8 correspond to the PB66 + PB67 primer pair, targeting exons 8–10. The results obtained with PB271 + PB272 and PB273 + PB274 are shown in Supplementary Fig. 8. Amplification was quantified in real time using SYBR-Green Master Mix in a Lightcycler LC480-II System (Roche, CA). After 40 cycles, the specificity of the amplification was verified by melting curve analysis, and the amplicon size was subsequently confirmed by electrophoresis in 2% (w/v) agarose gels. All reactions were performed in duplicate. Expression levels were calculated using the 2^−∆∆Ct^ method combining both *GAPDH* and *TPT1* as normalization genes. In all PCR reactions, experimentally determined amplification efficiency was very close to 2 within the range of concentrations assayed. A GFF3 file with the exact location of the primers and relevant sequence features is provided as Supplementary material (Features.gff3).

### Data from the The Cancer Genome Atlas (TCGA)

Clinical DNA methylation and mRNA expression data from TCGA CRC dataset (COAD + READ datasets)^17^ was downloaded from the International Cancer Genome Consortium (ICGC) website^39^. For the comparative analysis of *VWA2* expression in other cancer types, log2 RSEM data (RNA-Seq by Expectation Maximization)^40^ was obtained from the Broad Institute GDAC Firehose server (http://gdac.broadinstitute.org/) using the FirebrowseR R package^41^.

### Data from the ENCODE project

c-myc ChIP-seq data was obtained from the ENCODE project^23^ and visualized on the UCSC genome browser^42,43^. Twenty two ChIP-seq experiments were available, performed on 9 cell lines: A549 (lung carcinoma, accession ENCSR000DYC), GM12878 (lymphoblastoid, accession ENCSR000DKU), H1-hESC (human embryonic stem cell, accession ENCSR000EBY and ENCSR000DLI), HELA S3 (cervix adenocarcinoma, accession ENCSR000DLN and ENCSR000EZD), HepG2 (hepatocellular carcinoma, accession ENCSR000DLR), K562 (myeloid leukemia, accession ENCSR000DLZ, ENCSR000EZV, ENCSR000EGJ, ENCSR000EZU, ENCSR000EGS, ENCSR000FAZ and ENCSR000FAG), MCF10A (mammary epithelial cells, accession ENCSR000DOM and ENCSR000DOS), MCF-7 (breast cancer, accession ENCSR000DMM, ENCSR000DMQ, ENCSR000DMP and ENCSR000DMJ), and NB4 (mouse neuroblastoma, accession ENCSR000EHR).

### Statistical analyses

Statistical analyses were performed in R environment for statistical computing^44^, using Rstudio integrated development environment^45^. Contingency tables were analyzed using Fisher’s exact test or chi-squared test. Continuous variables were first tested for normality using Shapiro-Wilk normality test. If the variable exhibited a normal distribution, Student’s t-test was applied. Otherwise, Wilcoxon signed-rank sum test was applied. In this work we have employed the following packages, available from the CRAN or Bioconductor^46^: mixtools^47^, Biostrings^48^, exactRankTests^49^, FirebrowseR^41^ and biomaRt^50,51^. Gene set enrichment analysis was performed using GSEA v2.0 from the Broad Institute^52^, applying the GSEA preranked method with default parameters on the list of genes ranked according to their correlation with *VWA2* expression.

## Electronic supplementary material

Supplementary Figures

Supplementary tables

Genetic features
