# Supplementary material for: Epigenetic and transcriptional dysregulation of VWA2 associated with a MYC-driven oncogenic program in colorectal cancer
Source: Sci Rep. 2018 Jul 23;8:11097. doi: 10.1038/s41598-018-29378-7 (PMC6056412; doi:10.1038/s41598-018-29378-7)
Supplement: Supplementary file 1 — Supplementary Figures [file 41598_2018_29378_MOESM1_ESM.pdf]

Epigenetic and transcriptional dysregulation of *VWA2* associated with a *MYC*-driven oncogenic program in colorectal cancer

Beatriz González, Ferran Fece de la Cruz, Johanna K. Samuelsson,

Andreu Alibés and Sergio Alonso

SUPPLEMENTARY FIGURES

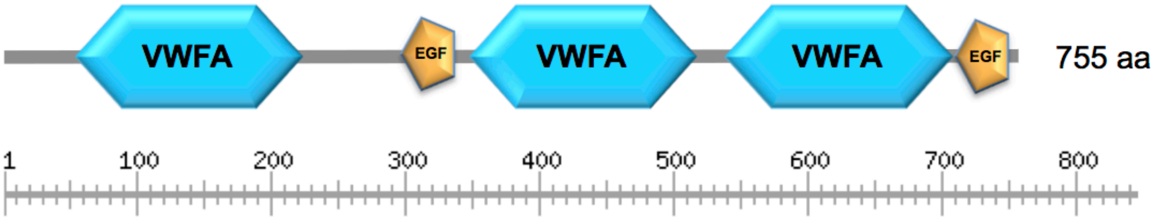

Supplementary figure S1. Protein domains of AMACO reveal its extracellular localization. The protein has a length of 755 aas, of which residues 1-23 correspond to the signal peptide. AMACO contains three VWFA domains typical of protein-protein interaction in extracellular matrix proteins (prosite PS50234, in blue), and two EGF-like domains (prosite PS50026, in orange) generally found in the extracellular domain of membrane-bound proteins or in secreted proteins.

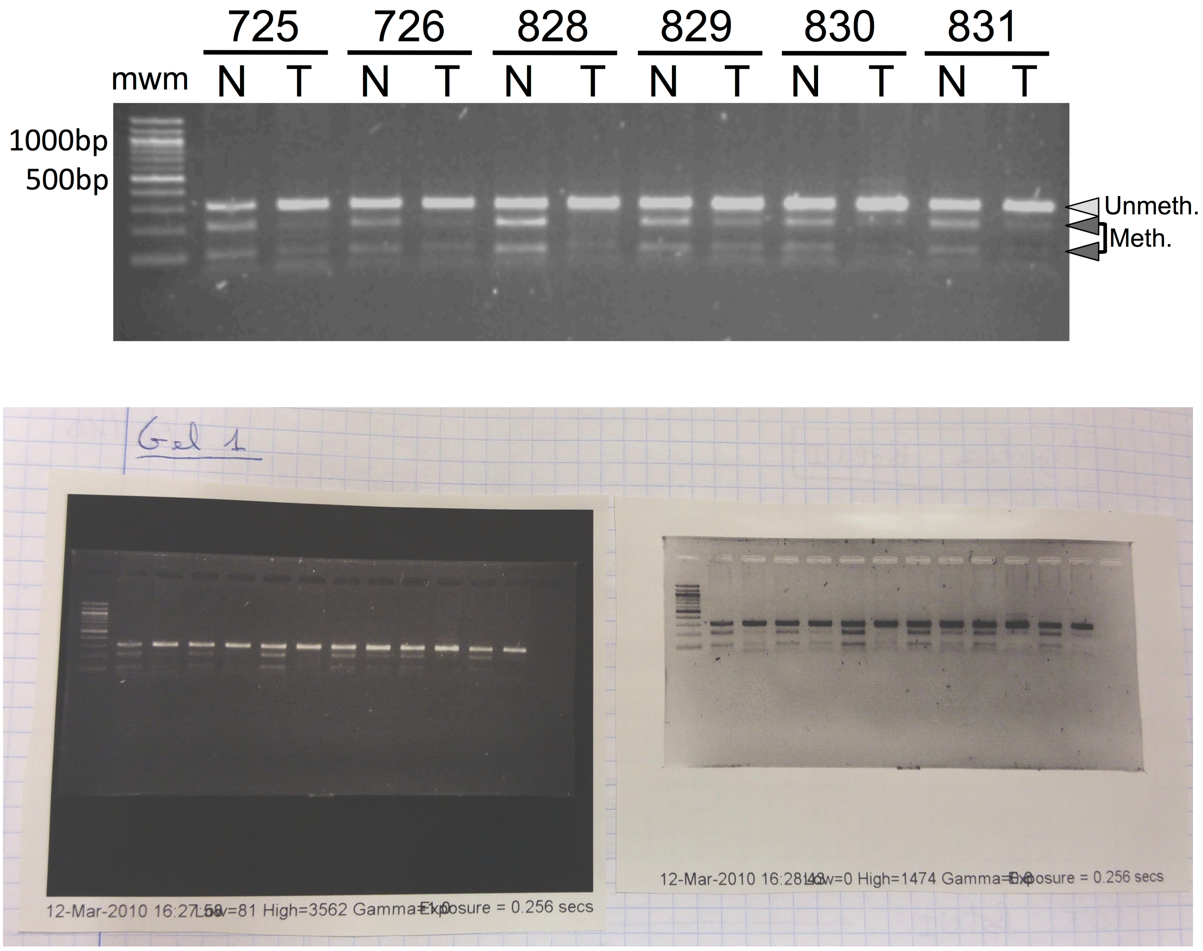

Supplementary figure S2. Upper panel: <u>*Bst*</u>UI-based **c**ombined bisulfite and restriction analysis (COBRA) of the *VWA2* region 4 in in normal (N) and tumor samples (T) from 6 CRC patients. This region contains a single *Bst*UI site that is digested in the PCR products of the originally methylated molecules, generating two restriction fragments of 219 and 96 bp (Meth., dark grey arrows). The PCR products of the unmethylated molecules (and heterodimers) are resilient to digestion, and are visualized as a single electrophoretic band of 315bp (Unmeth., light grey arrow). mwm is a 100bp ladder molecular weight marker. Somatic hypomethylation is reflected by lower intensity of the restriction fragments in tumors compared to the matched normal samples. Lower panel: Original pictures of the agarose gel directly taken from the BioRad Gel Doc system, with original (left) and inverted (right) color.

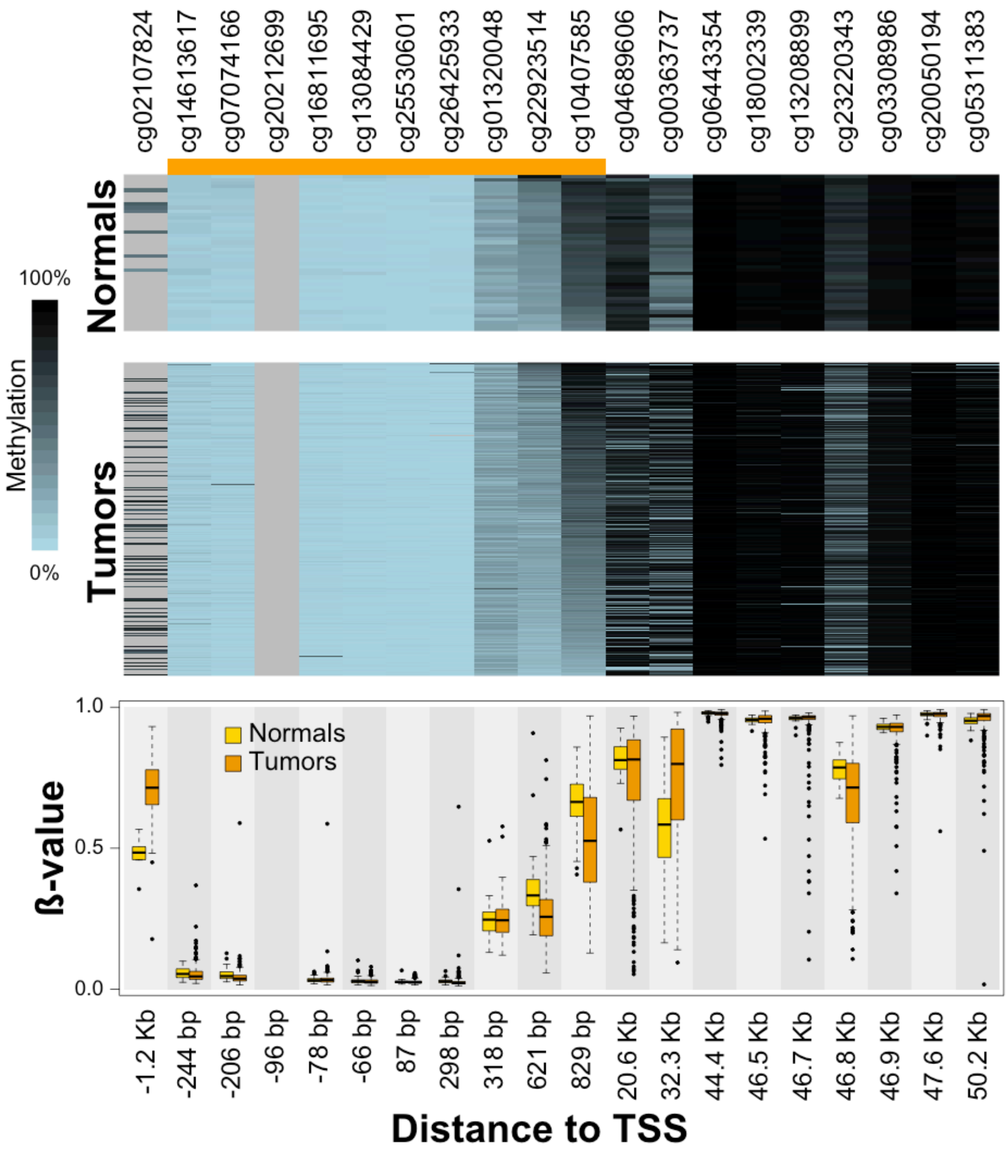

Supplementary figure S3. (top) Heatmap of the methylation of probes within the *VWA2* locus in 45 normal tissues and 352 tumor tissues from CRC patients (data from the TCGA). Methylation level is represented in a blue to black gradient. In grey, values that have been filtered by the TCGA analysis pipeline. The orange bar on top of the heatmap indicates probes within the ±1Kb of the TSS. (Bottom) Boxplot of the methylation, in ß-values, of these probes in normal tissues (yellow) and tumors (orange). The distance to the transcriptional start site (TSS) is indicated in the x-axis.

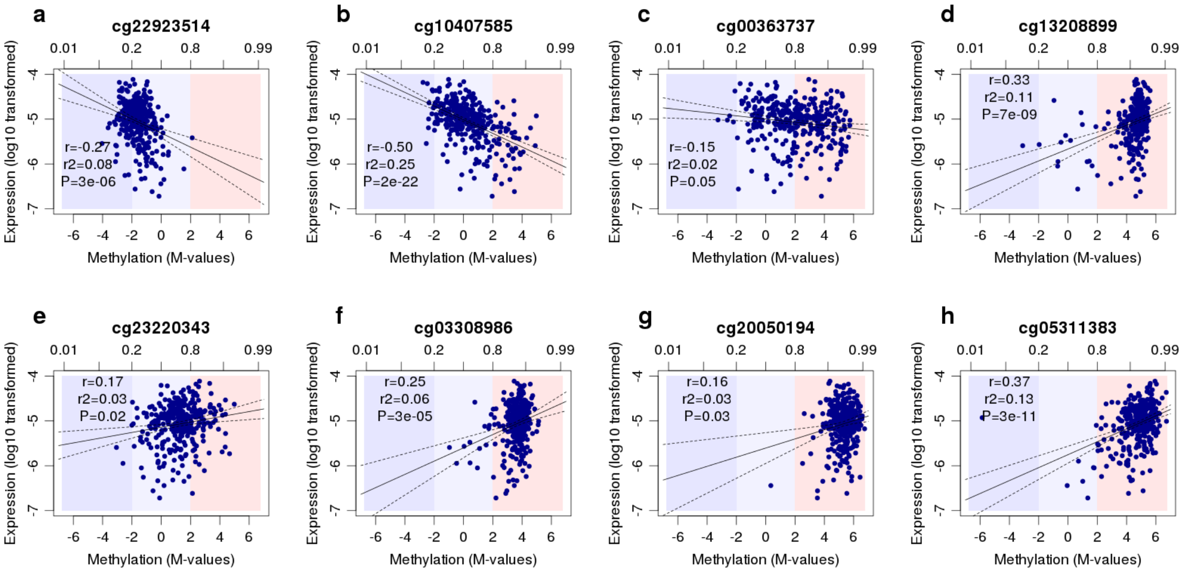

Supplementary figure S4. Methylation of HM450K probes (x-axis, represented as M-values) vs *VWA2* expression (y-axis, log10-transformed normalized read counts) in 348 CRC tumors from the COAD and READ datasets from the TCGA. Only probes within the *VWA2* locus and with P-values < 0.05 are shown. Pearson's product-moment correlation coefficient (r), coefficient of determination (r^2) and P-values after multi-hypothesis testing correction are indicated for every probe. M-values of -2 and 2 correspond to ß-values of 0.2 and 0.8, respectively, commonly considered thresholds for demethylation (darker blue areas) and full methylation (pink areas). Regression lines (solid) and their 95% confidence interval (dashed lines) are represented for every studied probe. The strongest association was found for probe cg10407585 (panel b, r^2=0.25), located in the south shore of the *VWA2* CGI.

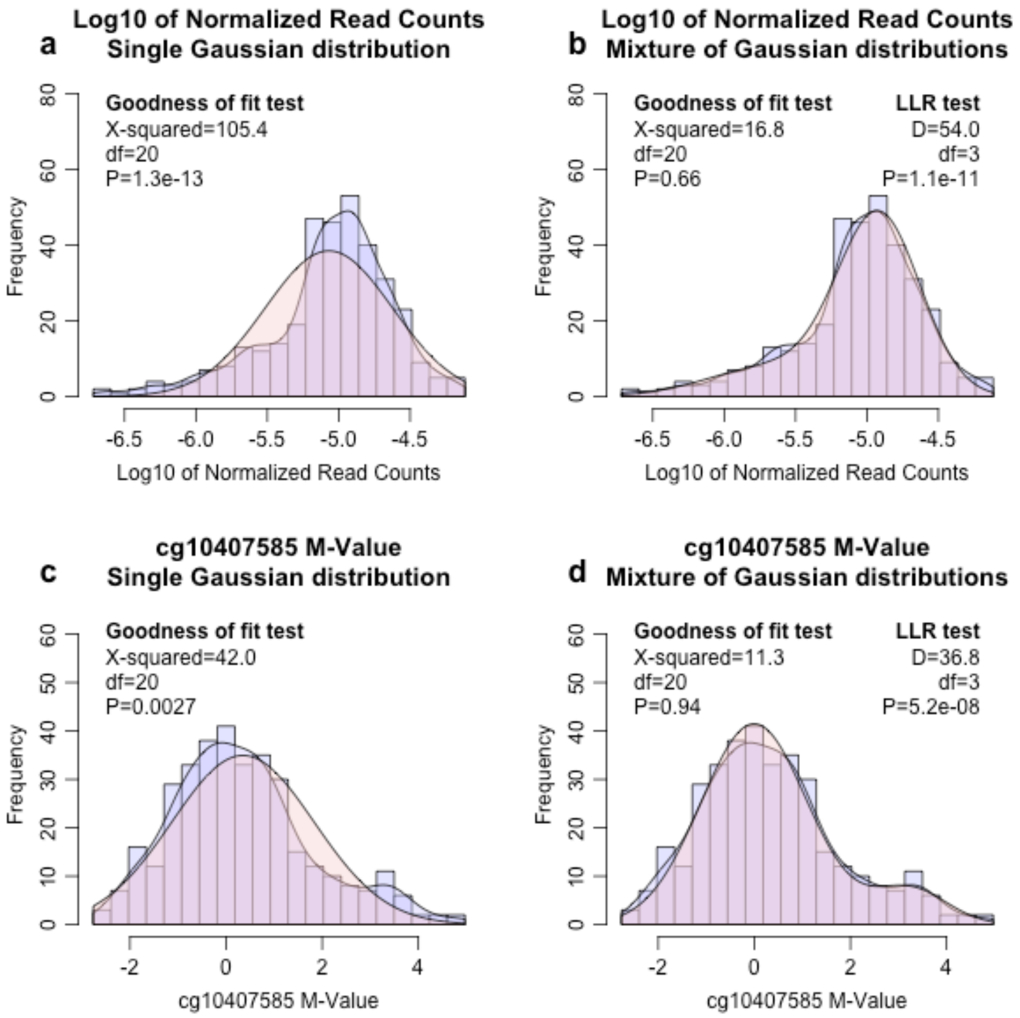

Supplementary figure S5. Modeling of *VWA2* expression (panels a and b) and cg10407585 methylation (panels c and d) in CRC tumor samples from the TCGA RNAseq and Illumina HM450K methylation datasets, respectively. Blue bars indicate the observed frequency. Blue-shaded areas represent the observed density. Pink-shaded areas represent the expected density under the different models, either single Gaussian distribution (a and c) or a mixture of 2 Gaussian distributions (b and d). Every panel includes a χ^2-based goodness-of-fit test (df: degrees of freedom, P: the P-value). Single models vs mixture models were compared by log-likelihood ratio test (LLR test) where D=2 x ln(likelihood$_{mixture\ model}$ / likelihood$_{single\ model}$). The comparison between models in a and b is shown in panel b. The comparison between models in c and d is shown in panel d. In both cases, the fit was greatly improved when mixtures of 2 Gaussian distributions were considered.

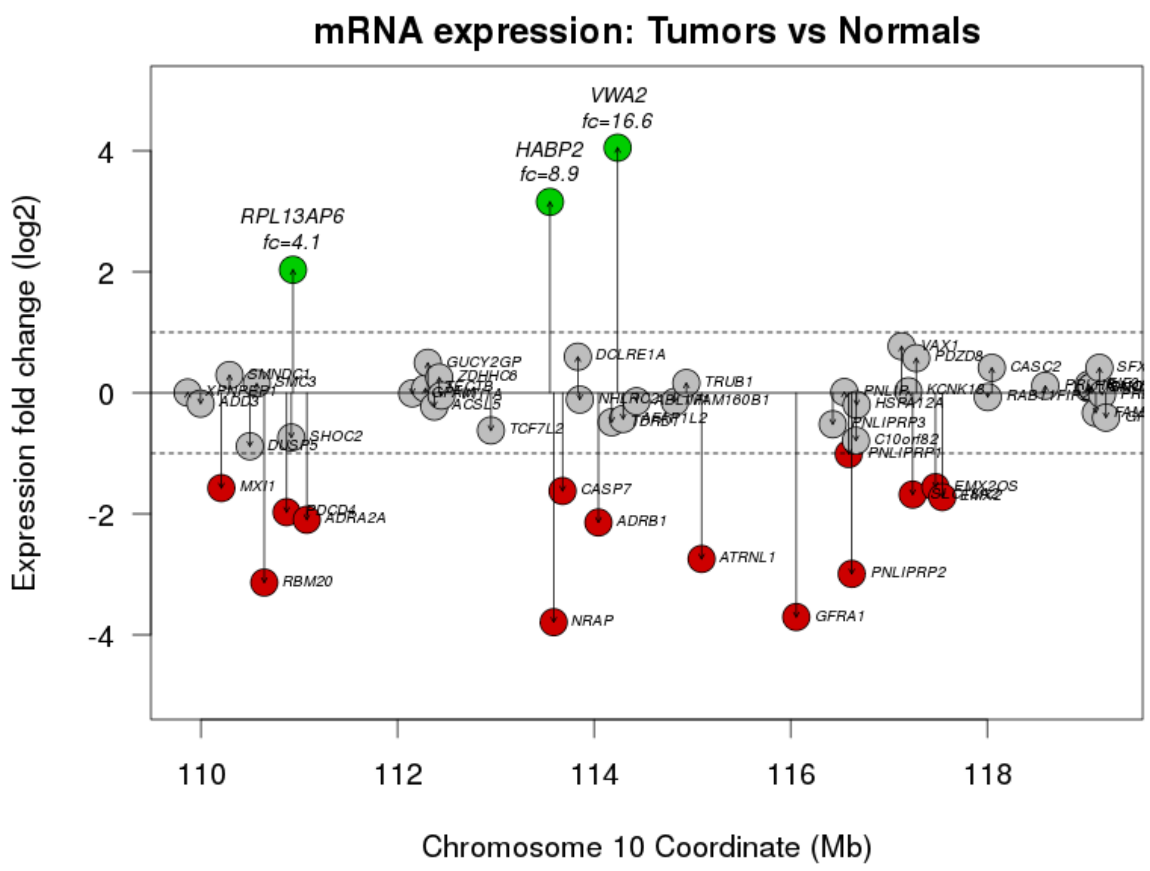

Supplementary figure S6. Transcriptional differences between CRC tumors (n=594) and normal tissues (n=50) in the 10Mb chromosome region surrounding *VWA2* locus. Data was obtained from the COAD and READ cohorts of the TCGA. Every dot represents a gene (n=55). In green, up-regulated genes (fold change larger than 2). In red, down-regulated genes (fold change lower than 0.5). For the three up-regulated genes, the fold change (fc) is shown.

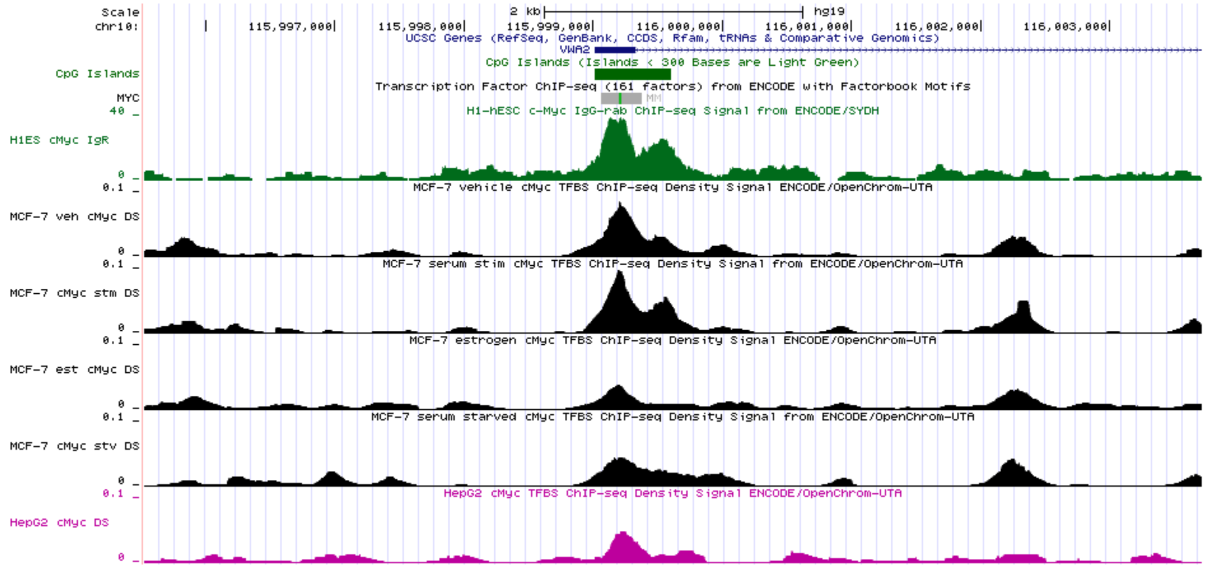

Supplemntary figure S7. c-myc ChIP-seq data from the ENCODE project, visualized in the UCSC genome browser. Samples with positive enrichment of c-myc at the first exon of *VWA2* are shown. From top to bottom: first track, in dark blue, the first exon of *VWA2*. Second track, in dark green, the promoter associated CpG island. Third track, in grey, the c-myc binding region determined by ChIP-seq experiments of the ENCODE project. The light green vertical bar within the grey rectangle indicates the E-box hexamer. Fourth track, in dark green, c-myc ChIP-Seq enrichment peaks from human embryonic stem cells H1-Esc, by the ENCODE/SYDH project. Tracks 5 to 8, in black, c-myc ChIP-Seq enrichment peaks from breast cancer MCF-7 cells, under different culture conditions: vehicle (veh), serum stimulated (stm), estrogen stimulated (est) and starved (stv). Ninth track, in purple, c-myc ChIP-Seq enrichment peaks from hepatocellular cancer HepG2 cells. Note that the binding of c-myc on the first exon of *VWA2* perfectly overlaps with the E-box located within its promoter associated CpG island.

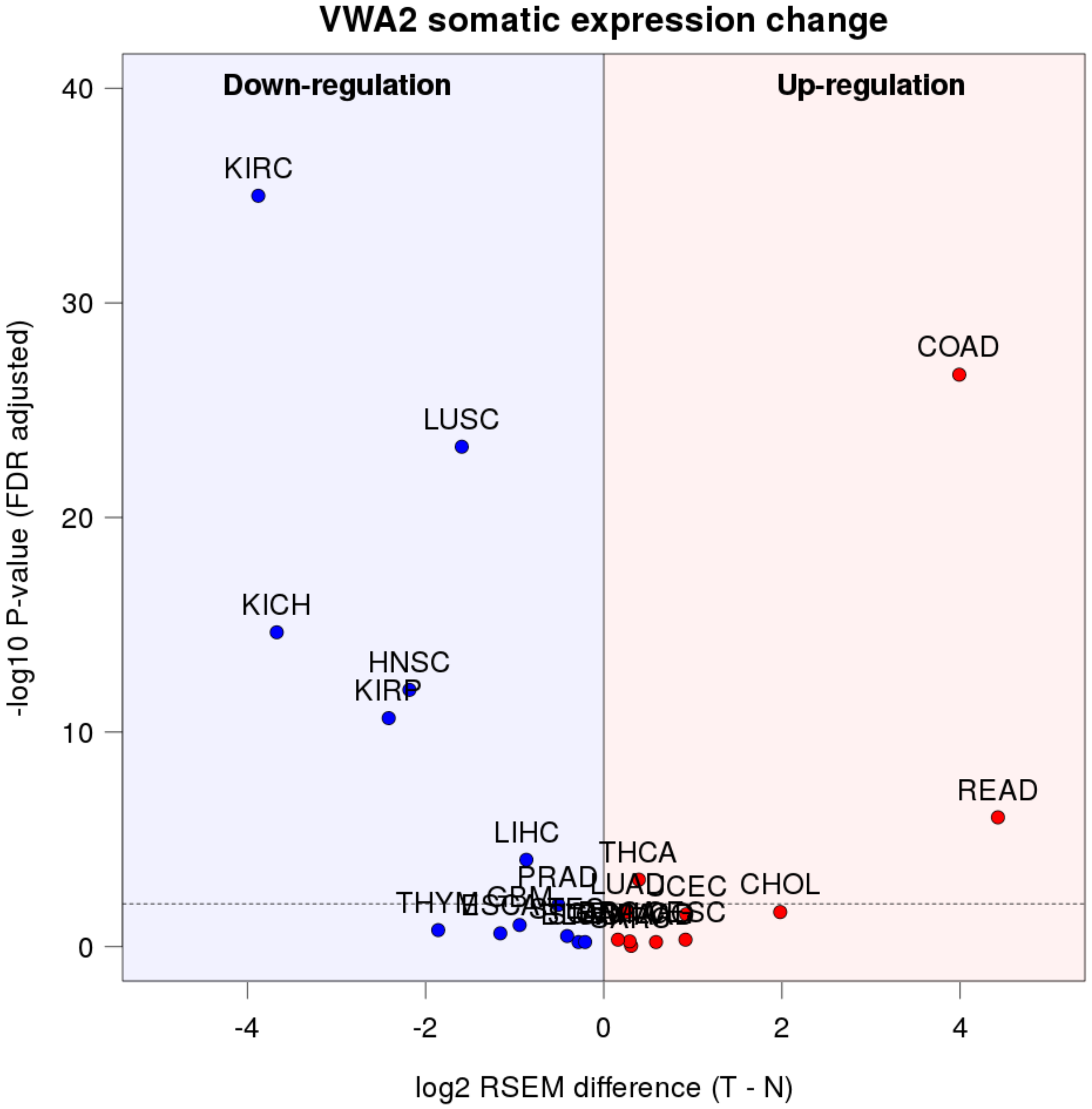

Supplementary figure S8. *VWA2* transcriptional expression difference between tumor and normal samples from 34 different cancer cohorts of the TCGA. In the x-axis, the difference in expression (logarithmic scale). In the y-axis the –log10 of the *P*-value, calculated by Student's t-test (see supplementary table ST7) and adjusted for multihypothesis testing using the FDR method. The horizontal dashed line indicates the statistical significance threshold (P=0.01). In blue, cancer types where VWA2 is down-regulated in tumors. In red, cancer types where VWA2 is up-regulated in tumors.

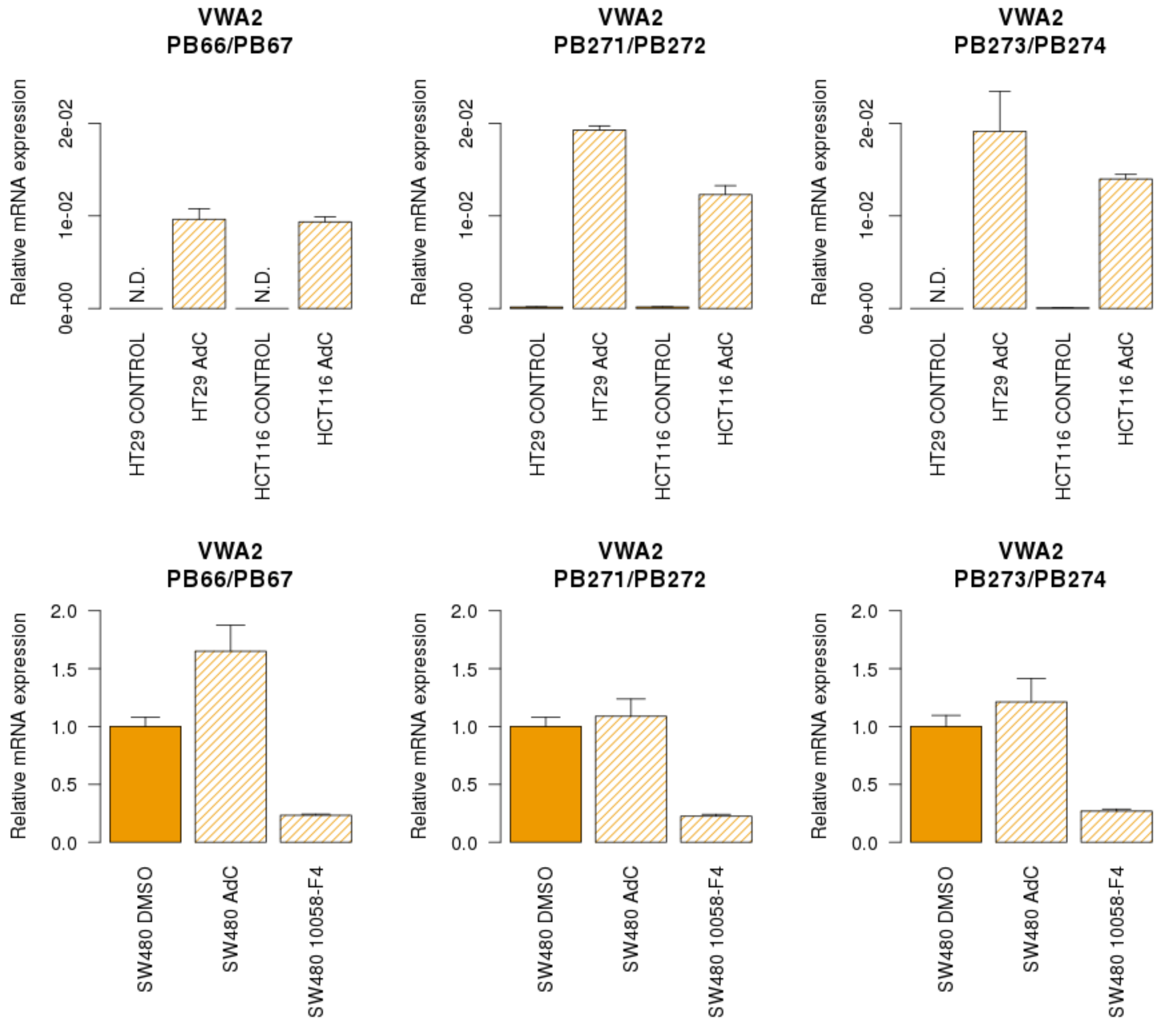

Supplementary figure S9. mRNA *VWA2* quantification by QPCR using primers targeting exons 8-10 (PB66/PB67), and exons 2-4 (PB271/PB272 and PB273/274). In the upper row, non-expressing cell lines HT29 and HCT116 treated with vehicle (control) or with 1μM 5-AZA-2-deoxycytidine (AdC) for 48h. N.D. non-detected. In the bottom row, highly expressing cell line SW480 treated with vehicle (DMSO), 1 μM AdC, or 100 μM 10058-F4 (and inhibitor of Myc/Max). All reactions were performed in duplicate and normalized with the housekeeping genes *GAPDH* and *TPT1*. Values were the scaled relative to the value of SW480 for every particular primer combination.
